# Macroecological patterns of archaeal ammonia oxidizers in the Atlantic Ocean

**DOI:** 10.1111/mec.13365

**Published:** 2015-09-28

**Authors:** Eva Sintes, Daniele De Corte, Natascha Ouillon, Gerhard J. Herndl

**Affiliations:** ^1^ Department of Limnology and Bio‐OceanographyCenter of EcologyUniversity of ViennaAlthanstrasse 141090ViennaAustria; ^2^ Department of Biological OceanographyRoyal Netherlands Institute for Sea ResearchPO Box 591790Den BurgThe Netherlands

**Keywords:** ammonia oxidizers, biogeography, deep ocean, macroecology, Thaumarchaeota

## Abstract

Macroecological patterns are found in animals and plants, but also in micro‐organisms. Macroecological and biogeographic distribution patterns in marine Archaea, however, have not been studied yet. Ammonia‐oxidizing Archaea (AOA) show a bipolar distribution (i.e. similar communities in the northernmost and the southernmost locations, separated by distinct communities in the tropical and gyral regions) throughout the Atlantic, detectable from epipelagic to upper bathypelagic layers (<2000 m depth). This tentatively suggests an influence of the epipelagic conditions of organic matter production on bathypelagic AOA communities. The AOA communities below 2000 m depth showed a less pronounced biogeographic distribution pattern than the upper 2000 m water column. Overall, AOA in the surface and deep Atlantic waters exhibit distance–decay relationships and follow the Rapoport rule in a similar way as bacterial communities and macroorganisms. This indicates a major role of environmental conditions in shaping the community composition and assembly (species sorting) and no, or only weak limits for dispersal in the oceanic thaumarchaeal communities. However, there is indication of a different strength of these relationships between AOA and Bacteria, linked to the intrinsic differences between these two domains.

## Introduction

The application of ecological theories to microbial organisms has gain renewed interest in the last decade (Martiny *et al*. 2006; Ramette & Tiedje 2007; Fierer 2008; Amend *et al*. 2013; van der Gast 2015). Although there are some singularities of the microbial ecology patterns due to differences in scale and physiology between macro‐ and micro‐organisms (Fierer *et al*. 2011; Carbonero *et al*. 2014), many macroecological patterns, yet quantitatively different, also extend to Bacteria (Soininen 2012; Amend *et al*. 2013). Patterns found in macroorganisms, such as taxa–area relationships and distance–decay patterns (Green *et al*. 2004; Horner‐Devine *et al*. 2004; Bell 2010; Astorga *et al*. 2012; Wetzel *et al*. 2012; Zinger *et al*. 2014), latitudinal species richness gradients (Pommier *et al*. 2007; Fuhrman *et al*. 2008) and the Rapoport rule (Amend *et al*. 2013; Sul *et al*. 2013) have been also reported for micro‐organisms.

In this context, the existence of biogeographic patterns for microbes is now widely accepted (Martiny *et al*. 2006; Ramette & Tiedje 2007; Fierer 2008). However, there is an intense debate on the underlying factors for these patterns, both for macro‐ and micro‐organisms. Are the observed patterns generated via selective processes, such as evolutionary adaptation and sorting of species according to the environmental conditions, or nonselective as proposed by neutral theory, such as dispersal and drift (Vellend *et al*. 2014)? This debate in microbial biogeography has been centred in the last decades on the Baas‐Becking principle ‘everything is everywhere, but, the environment selects’ (Baas‐Becking 1934). Recent research argues that the generalizing ubiquitous dispersal hypothesis highlighted by this tenet should be rejected (van der Gast 2015) and the relative influence of selective (e.g. niche suitability) and nonselective (stochastic, historical) processes should be considered and disentangled (Barberan *et al*. 2014).

Although the dark ocean harbours 75% of the prokaryotic biomass (Arístegui *et al*. 2009), macroecological patterns of microbes, including biogeographic studies, have focused mostly on Protozoa (Finlay & Fenchel 2004; Foissner 2006) and epipelagic bacterial communities (Pommier *et al*. 2007; Sul *et al*. 2013) with few exceptions (Ghiglione *et al*. 2012). Generally, only limited information is available on the archaeal communities inhabiting the deep ocean. There are intrinsic differences between bacterial and archaeal domains such as their cell wall, metabolic pathways, molecular repertoire and diversity (DeLong & Pace 2001; Verhees *et al*. 2003; Wang *et al*. 2007; Ellen *et al*. 2010; Albers & Meyer 2011; Yarza *et al*. 2014) that could influence both selective processes and dispersal. Only recently, however, archaeal biogeography has been studied across different environments (from symbionts to terrestrial and aquatic habitats) based on 16S rRNA (Auguet *et al*. 2010) and *amo*A sequences (Fernández‐Guerra & Casamayor 2012; Cao *et al*. 2013), or focusing on aquatic ecosystems (Biller *et al*. 2012) or soils (Pester *et al*. 2012). These studies focused on differences between habitats. Thus, macroecological patterns, such as distance‐decay or latitudinal range, were not inferred for Archaea in contrast to studies on Bacteria (Sul *et al*. 2013).

The goal of this study was to provide a comprehensive view on the macroecological patterns of ammonia‐oxidizing Archaea (AOA) along a latitudinal gradient and throughout the water column of the Atlantic Ocean. Our hypothesis was that the intrinsic lower diversity of Archaea, in particular Thaumarchaeota, together with other intrinsic differences such as differential dispersal rates associated to different population size, etc., results in different latitudinal and depth‐related patterns in Archaea as compared to Bacteria and Eukarya.

## Materials and methods

Sampling was conducted during the GEOTRACES‐1 and ‐2 cruises with R/V *Pelagia*, between April and June 2010 and GEOTRACES‐3 onboard of R/V *James Cook* between February and April 2011. Water samples were taken at 51 stations (Fig. S1, Supporting information) with 24 × 25L‐Niskin bottles mounted in a frame holding also sensors for conductivity–temperature–depth (CTD), salinity, oxygen, fluorescence and optical backscattering. Contextual environmental parameters, such as inorganic nutrients and microbial abundance, were analysed at 24 depth layers from surface to abyssopelagic waters as detailed in Appendix S1 (Supporting information). Samples for the analyses of AOA were collected at 6–8 depths from the euphotic layer (50 m depth), the oxygen minimum zone and mesopelagic (<1000 m), upper bathypelagic (1000–2000 m) and lower bathypelagic and abyssopelagic depths (>2000 m). Quantitative PCR (Q‐PCR) was used with slight modifications to evaluate 16S rRNA gene abundance of Thaumarchaeota and the abundance of two ecotypes of AOA based on their *amo*A gene: the low ammonia concentration ecotype (LAC‐AOA) and the high ammonia concentration ecotype (HAC‐AOA) as previously described (Sintes *et al*. 2013) (Appendix S1, Supporting information). Six different oceanographic regions were differentiated along this transect based on the description of Longhurst (2007): the North Atlantic Arctic province (ARCT; 70°N–55°N), the North Atlantic Drift province (NADR; 55°N–40°N), the North Atlantic Gyral province (NAG) comprising the North Atlantic Tropical and the Subtropical Gyral province (40°N–12°N), the Western Tropical Atlantic (WTRA; 12°N–6°S) province, the South Atlantic Gyral (SATL; 6°S–40°S) and the Subantarctic province (SANT) comprising the Subtropical Convergence Zone (SSCT; 40°S–45°S) and the Subantarctic Water Ring province (SANT; 45°S–55°S) (Fig. S1, Supporting information). GPS coordinates and sampling date for each station are available on Dryad (doi: 10.5061/dryad.c6688).

To evaluate the community composition of AOA, terminal‐restriction fragment length polymorphism (T‐RFLP) analysis of the archaeal *amo*A gene was conducted on all the samples after its amplification using specific primers as described in the Appendix S1 (Supporting information). Briefly, labelled primers cren amo_F‐FAM (Hallam *et al*. 2006) and amoAR‐VIC (Francis *et al*. 2005) were used to amplify archaeal *amo*A. FAM‐ and VIC‐labelled purified PCR products were digested at 37 °C overnight with three different restriction enzymes (*Mbo*I, *Rsa*I and *Hae*III). After separation of the labelled fragments with a 3130xL Genetic Analyzer capillary sequencer (Applied Biosystems), the electropherograms were analysed with GelComparII software (Bio‐Rad Laboratories). The threshold level to discriminate bands was set at 0.5% of the total peak height. The obtained matrix was analysed by Primer software (Primer‐E, Ltd, Ivybridge, UK) to determine the similarity between the different T‐RFLP patterns obtained from the samples. Standardized and normalized OTU abundance data obtained by T‐RFLP for the different samples are available on Dryad (doi: 10.5061/dryad.c6688).

Additionally, one sample from each oceanic province and depth layer (Table S1, Fig. S1, Supporting information) was chosen to generate *amo*A gene sequence libraries with Sanger sequencing and/or 454‐pyrosequencing (Appendix S1, Supporting information). 454‐pyrosequencing of archaeal *amo*A was performed at IMGM Laboratories GmbH (Germany) on a Roche 454 GS Junior platform based on titanium chemistry. All samples were barcoded using multiplex identifiers and sequenced together in one run. Raw 454 sequences were initially trimmed using lucy 1.20 (Chou & Holmes 2001) keeping sequences of ≥250 nt which had an average Phred score of ≥27. Subsequently, the remaining sequences were screened for the barcode and primer sequences keeping only the sequences that had exact matches.

The sequences selected by the above procedure were processed following a similar pipeline as described elsewhere (Pester *et al*. 2012). Briefly, sequences were preclustered using the pre.cluster function in MOTHUR (Schloss *et al*. 2009) with *n *= 3 (sequence identity ≥97.6% for sequences ≥250 nt). Representatives of the pre.cluster step were further grouped using the CD‐HIT‐454 (http://weizhong-lab.ucsd.edu/cd-hit/servers.php) clustering tool (Huang *et al*. 2010) at a 98.5% sequence identity level over 97% of the smaller sequence. Thereafter, HMMFrame (Zhang & Sun 2011) was used to screen possible frame shifts in representative sequences of all CD‐HIT clusters.

After manual chimera removal (Pester *et al*. 2012), sequences were grouped based on their sequencing direction (forward or reverse) and rarefaction curves, binning into OTUs, and α‐diversity analysis was conducted using MOTHUR (Schloss *et al*. 2009). The remaining sequences were aligned together with the clone sequences and NCBI reference sequences from *N. maritimus*,* Nitrososphaera gargensis*,* Nitrosoarchaeum limnia* and *Cenarchaeum symbiosum* to infer their phylogeny. Raw 454‐pyrosequences of *amo*A have been deposited in NCBI, Accession no. SRP049002. A detailed description of the pyrosequencing approach can be found in the Appendix S1 (Supporting information). AOA OTUs abundance data obtained by pyrosequencing are available on Dryad (doi:10.5061/dryad.c6688).

AOA OTUs were assigned as the gene sequences sharing 98% identity (Agogué *et al*. 2008). Although this sequence similarity of *amo*A gene might not correspond to different archaeal species (Pester *et al*. 2012), it was chosen to keep the information on the diversity of the functional group. Although this selection might increase the diversity values obtained for Thaumarchaeota, it will facilitate observing patterns in the functional group of AOA.

T‐RFLP fingerprinting, Sanger sequencing and 454‐pyrosequencing analyses targeted the entire archaeal *amo*A harbouring community. T‐RFLP allowed a relatively fast analysis at high spatial resolution (in total, 295 samples were efficiently fingerprinted with this method), while 454‐pyrosequencing was used to attain an in‐depth phylogenetic assessment of the AOA community, including the low abundance groups. Additionally, the Sanger sequencing provided a high quality database of archaeal *amo*A sequences from the same stations where pyrosequencing was conducted, facilitating the identification of chimeras and the alignment of the pyrosequences. The role of environmental (temperature, depth, nitrite, oxygen concentration, latitude) and biological (Thaumarchaeota 16S rRNA, LAC‐ to HAC‐*amo*A ecotype abundance) factors on the AOA community composition (assessed either by T‐RLP or 454 pyrosequencing) was evaluated by canonical correspondence analysis (CCA). Partial redundancy analysis (partial RDA) was used to discriminate the contribution of different explanatory variables (environment, space and time) on the variation of the AOA community composition in the Atlantic (Appendix S1, Supporting information).

## Results

### Diversity of archaeal ammonia oxidizers: latitudinal and depth patterns

**Figure 1:**
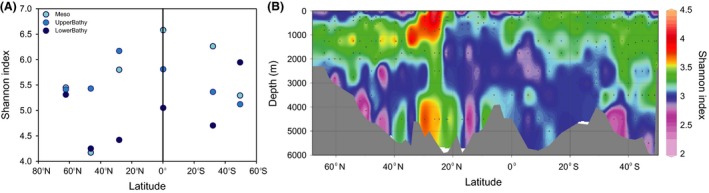
Diversity (Shannon index) of AOA communities in the mesopelagic (Meso), upper (UpperBathy) and lower bathypelagic layer (LowerBathy) in different regions obtained from the 454‐pyrosequencing libraries (A), and based on the depth profiles using T‐RFLP fingerprinting (B).

Rarefaction curves (Fig. S2, Supporting information) obtained for Sanger sequencing and 454‐pyrosequencing libraries indicated that the epipelagic AOA communities exhibited a lower richness than meso‐ and bathypelagic AOA communities of low latitude provinces (Table S2, Supporting information). A trend of increasing richness (Table S2, Supporting information) and diversity (Fig. 1A, Table S2, Supporting information) towards the gyral and tropical regions was evident from the mesopelagic libraries, while the highest richness and diversity of upper bathypelagic AOA communities were found in the NAG province. Richness and diversity of lower bathypelagic (below 2000 m depth) AOA communities were less variable than in the upper bathypelagic realm (1000–2000 m depth) and were highest in the ARCT, WTRA and SANT provinces (Table S2, Figs 1A and S2, Supporting information). Slight differences (compare Figs 1A and B) in the location of the diversity maximums between pyrosequencing and T‐RFLP fingerprinting approaches (i.e. maximum for meso‐ and lower bathypelagic communities at the equator and around 30°N, respectively) might be caused by the different detection levels of the two methods.

T‐RFLP fingerprinting of the AOA revealed distinct communities associated to the oceanographic provinces and depth layers (two‐way ANOSIM, *R* = 0.35 and *R* = 0.42, respectively, *P* = 0.001). The Shannon index of diversity obtained from T‐RFLP fingerprints was generally higher for mesopelagic and upper bathypelagic waters (Fig. 1B) than for lower bathypelagic layers, in agreement with results obtained by cloning and pyrosequencing (Table S2, Supporting information, Fig. 1A). The highest diversity indexes were obtained for the oligotrophic gyral provinces (Fig. 1B).

Considering the communities of different depth layers separately (Fig. S3, Supporting information), AOA communities assessed by T‐RFLP fingerprinting grouped according to oceanic provinces, especially in epi‐ and mesopelagic waters (Fig. S3A and B, Supporting information, respectively). AOA from the ARCT, NADR and SANT provinces tended to cluster together (Fig. S3, Supporting information) and shared a higher number of OTUs among them as assessed by T‐RFLP and 454 pyrosequencing than with gyral and tropical provinces (Table S3, Supporting information, Fig. 2A, B). In contrast, AOA communities of the NAG, WTRA and SATL provinces shared a higher number of AOA OTUs (Table S3, Supporting information, Fig. 2C, D) than with high latitude AOA communities. This trend resulted in a decrease in similarity between AOA communities (determined by T‐RFLP fingerprinting) from ~50°S to 0°, followed by an increase in similarity towards 64°N (Fig. 3). This latitudinal trend in similarity of AOA communities was more pronounced in epipelagic waters (*r *= 0.80, *P* < 0.0001, Fig. 3), than for the mesopelagic (*r *= 0.68, *P* < 0.0001) and upper bathypelagic AOA communities (*r *= 0.66, *P* < 0.0001). The similarity between AOA communities was higher and less variable in upper bathypelagic waters than in meso‐ and epipelagic layers (Fig. 3). In contrast to the AOA communities of the upper water layers, the AOA communities in the lower bathypelagic layer showed no significant relation between similarity in community composition and distance (*r *= 0.41, *P* > 0.4).

**Figure 2:**
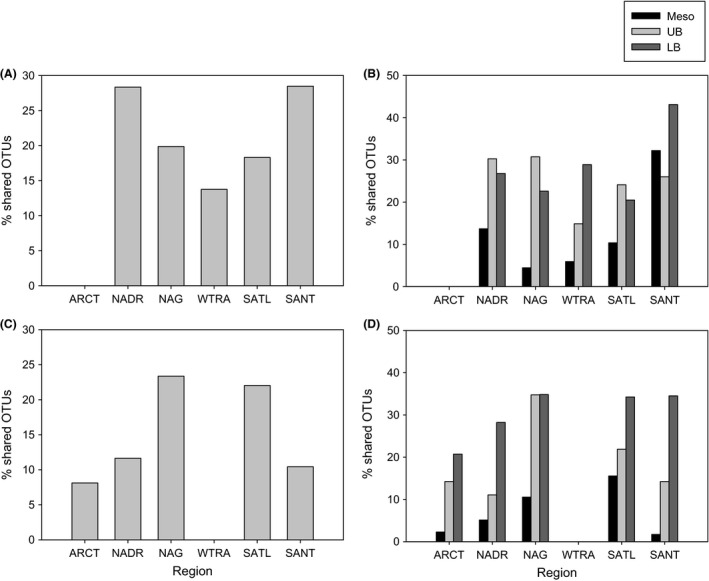
Proportion of shared OTUs of the ARCT region with all the other regions: for the whole water column (A) and for mesopelagic (Meso), upper (UB) and lower (LB) bathypelagic layers (B) separately. The proportion of OTUs from the WTRA region shared with other regions in the Atlantic for the entire water column (C) and for distinct water layers (D).

**Figure 3:**
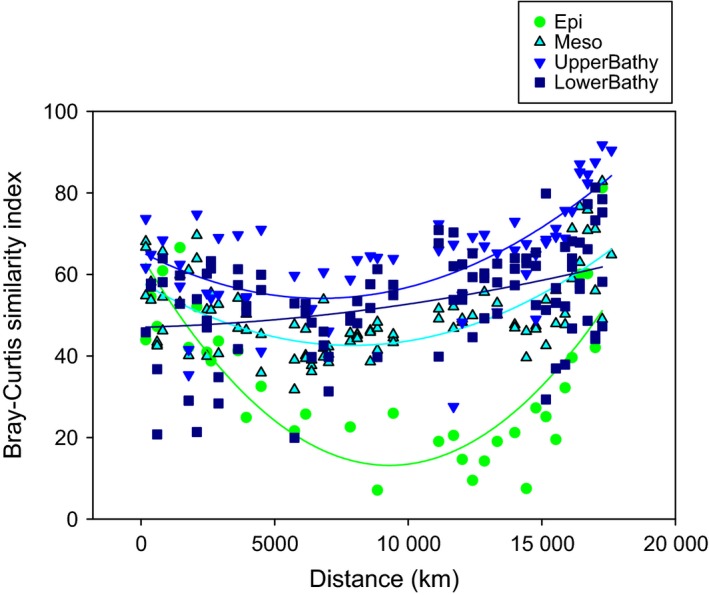
Pairwise similarity between AOA community fingerprints *vs*. distance (km) for the epipelagic (Epi), mesopelagic (Meso), upper (UpperBathy) and lower bathypelagic (LowerBathy) throughout the Atlantic Ocean, all related to the southernmost station. Each point represents the Bray–Curtis similarity index between the community composition from a specific depth layer in the southernmost station and the community composition at the same depth layer from a station located at a distance x.

**Figure 4:**
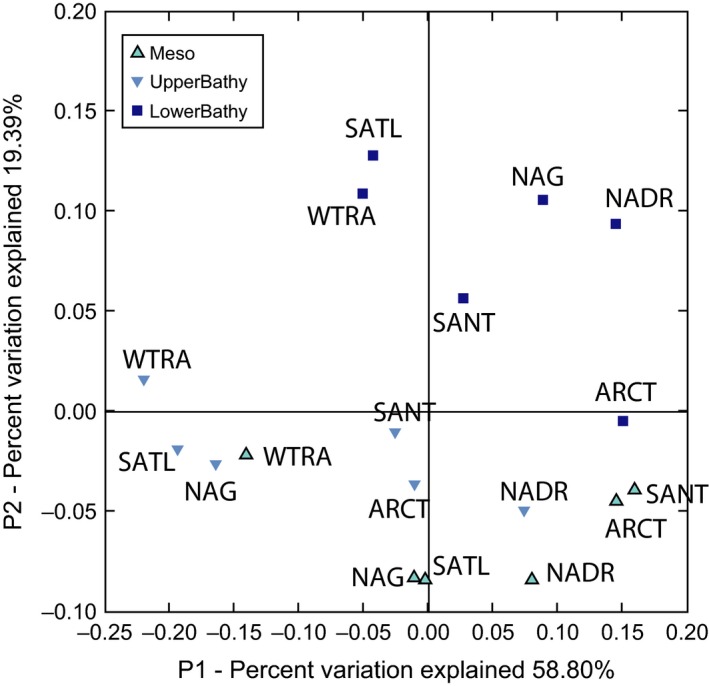
Principal coordinates analysis of the 18 forward‐pyrosequenced archaeal *amo*A libraries. Different colours denote depth layers (Meso: mesopelagic, UpperBathy: upper bathypelagic, LowerBathy: lower bathypelagic), oceanographic regions are noted next to the symbols, for abbreviations see legend of Fig. S1 (Supporting information).

The latitudinal tendency in the similarity of the AOA communities was also apparent from the archaeal *amo*A pyrosequencing libraries, exhibiting a higher similarity between ARCT and SANT communities and between NAG and SATL communities (Fig. 4) than to the AOA communities of other provinces, even though they are farther apart than, for example ARCT from NAG, or NAG from WTRA (Fig. 4).

In regions of deep‐water mass formation or upwelling (ARCT, WTRA and SANT, Fig. S4C, Supporting information), the different depth layers shared a higher number of OTUs (Fig. S4A, B, D, Supporting information) than in the stratified water column of the NADR and the gyral provinces (Fig. S4A, B, D, Supporting information).

### Latitudinal and temperature range of individual members of the AOA community

**Figure 5:**
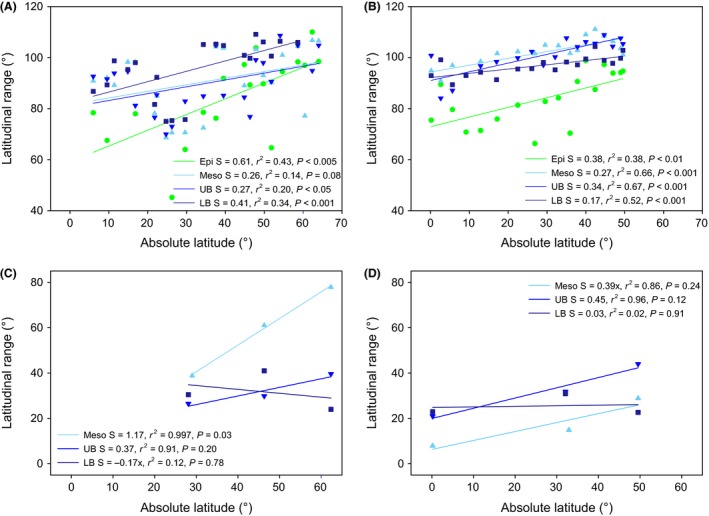
Relationship between AOA average latitudinal range size and latitude. Each symbol represents the average latitudinal range for a sample, based on T‐RFLP OTUs for the (A) northern hemisphere, (B) southern hemisphere of the Atlantic, and based on 454 pyrosequences for the northern (C) and southern (D) hemispheres. Different symbols denote the different depth layers: epipelagic (Epi), mesopelagic (Meso), upper (UB) and lower (LB) bathypelagic.

As revealed by both T‐RFLP and pyrosequencing, AOA OTUs from all depth layers and both hemispheres followed the Rapoport rule, that is OTUs from high latitudes exhibited a broader latitudinal range than OTUs from low latitudes (Fig. 5), except the lower bathypelagic OTUs obtained by pyrosequencing (>2000 m depth, Fig. 5C, D). Our results also indicate a broader latitudinal range of abundant OTUs than of less abundant OTUs (Table S4, Supporting information). In terms of temperature range of AOA OTUs, only epipelagic OTUs and surprisingly, upper bathypelagic (1000–2000 m depth) AOA OTUs exhibited a significant temperature range that mimicked the latitudinal range (Fig. S5, Supporting information).

### Environmental factors influencing the distribution of AOA

**Figure 6:**
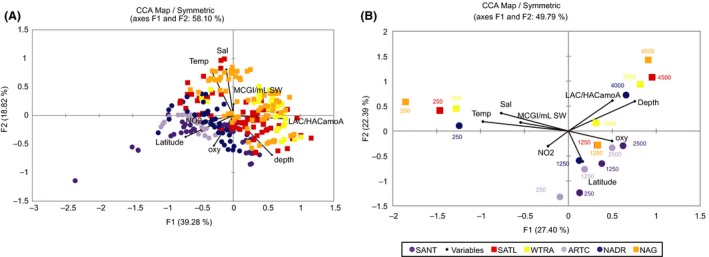
Canonical correspondence analysis (CCA): for (A) T‐RFLP fingerprints of the forward region, and (B) 454‐pyrosequencing libraries sequenced with the forward primer. Symbols represent different oceanic provinces, for abbreviations see legend of Fig. S1 (Supporting information). Next to the symbols, the depth of the individual sample is indicated. Arrows indicate selected environmental variable (Latitude, Depth, Sal: salinity, Temp: temperature, oxy: dissolved oxygen concentration, NO2: nitrite concentration, MCGI/mL SW: 16S rRNA gene abundance of Thaumarchaeota, LAC/HACamoA: ratio between LAC and HAC‐*amo*A gene).

The role of environmental factors on the AOA community composition was assessed by CCA. The first two coordinates explained 58.1% and 56.3% of the variation in the T‐RFLP fingerprints obtained from the forward and the reverse regions, respectively (Figs 6A and S6A, Supporting information). Coordinate 1 was positively related to the ratio LAC‐*amo*A to HAC‐*amo*A and negatively to latitude and nitrite concentration, while coordinate 2 was positively related to temperature, salinity and abundance of Thaumarchaeota 16S rRNA genes and negatively to depth and, to a lesser extent, to oxygen concentration.

Using *amo*A pyrosequence data, 49.8% and 47.6% of the variation was explained by the first 2 coordinates for the forward‐ and reverse‐sequenced *amo*A, respectively (Figs 6B and S6B, Supporting information). Coordinate 1 was strongly negatively related to temperature, salinity and abundance of Thaumarchaeota (MCGI) 16S rRNA gene and positively to oxygen concentration for the forward‐sequenced *amo*A (Fig. 6B). The AOA communities from the oxygen minimum zone (250 m depth) are separated from the communities from other depth layers by coordinate 1 except the AOA community of the ARCT and SANT province. Coordinate 2 was positively related to depth and the ratio between low ammonia concentration (LAC‐) *amo*A ecotype and high ammonia concentration (HAC‐) *amo*A ecotype abundance and negatively to latitude and nitrite concentration. The AOA communities from ARCT and SANT are separated from the other regions by coordinate 2. Similar relationships, with reversed axes, were observed for the reverse‐sequenced *amo*A (Fig. S6B, Supporting information).

## Discussion

### Factors driving the distribution and diversity of AOA throughout the Atlantic

Several environmental variables have been identified as potentially important in regulating AOA community composition and distribution (Erguder *et al*. 2009), such as salinity (Francis *et al*. 2005; Mosier & Francis 2008; Sahan & Muyzer 2008; Abell *et al*. 2010), temperature (Biller *et al*. 2012), pH (Pester *et al*. 2012) in soils, nitrite (Herfort *et al*. 2007), dissolved oxygen (Santoro *et al*. 2008), light (Merbt *et al*. 2012), latitude (Biller *et al*. 2012; Pester *et al*. 2012) or depth (Biller *et al*. 2012), leading to the habitat‐phylogeny association of the microbial ammonia oxidizers (Fernández‐Guerra & Casamayor 2012). Our data indicate a strong influence of latitude and depth on the AOA community composition (Figs 6 and S6, Supporting information), but the underlying factors for this influence include nitrite concentration, temperature and dissolved oxygen concentration. Although it is not clear how temperature and oxygen concentration might affect AOA, it has been shown that temperature relates to archaeal *amo*A community composition (Biller *et al*. 2012). Also, Thaumarchaeota are frequently associated to oxygen minimum zones (Agogué *et al*. 2008; Beman *et al*. 2008), where ammonia is released by remineralization processes (Wuchter *et al*. 2006). This suggests an indirect effect of dissolved oxygen concentration on AOA community composition. Ammonium concentration has been suggested to determine the abundance and oxidation rates not only of ammonia‐oxidizing Bacteria (AOB), but also of AOA (Christman *et al*. 2011). Although we have no ammonium measurements, the end product of ammonia oxidation (${\text{NO}}_{2}^{-}$), and the ratio between the two ecotypes of AOA, which is linked to ammonia and nitrite concentrations (Sintes *et al*. 2013), strongly relates to the AOA community composition (Figs 6 and S6, Supporting information), supporting the significance of this nutrient in shaping the ammonia‐oxidizing community.

The variation of the AOA community was explained not only by environmental factors, but also by spatial and temporal factors, explaining altogether 44% as estimated by RDA (Appendix S1, Supporting information). Variation partitioning supported the main role of the spatial and environmental factors on the community composition (Fig. S7, Supporting information) as compared to the possible temporal effect associated to the different sampling times.

### Latitudinal ranges of archaeal ammonia oxidizers

AOA OTUs from high latitudes exhibit a wider latitudinal range than OTUs from low latitudes at all depth layers, except the lower bathypelagic OTUs determined by 454‐pyrosequencing (Fig. 5). This finding is in agreement with studies on bacterial taxa (Amend *et al*. 2013; Sul *et al*. 2013). The broader range in AOA diversity at high than at low latitudes follows the Rapoport rule (Stevens 1989), found in plants and animals though with many exceptions (Rohde *et al*. 1993; Gaston *et al*. 1998; Rohde 1999). Also, abundant OTUs exhibit a broader latitudinal range as compared to less abundant OTUs (Sul *et al*. 2013). The mesopelagic AOA communities, also following the Rapoport rule, do not show a relation between temperature range and temperature (Fig. S5, Supporting information). This suggests that both latitudinal distance (as an indicator of dispersal limitation) and current environmental conditions (an indicator of species sorting) determine mesopelagic archaeal biogeography (Sul *et al*. 2013).

### Bipolar distribution of archaeal ammonia oxidizers in the ocean

A distinct latitudinal gradient in alpha‐diversity of the AOA community was detectable (Fig. 1), with higher diversity in low latitude than in high latitude regions, similar to the diversity pattern reported for Bacteria (Pommier *et al*. 2007; Fuhrman *et al*. 2008; Sul *et al*. 2013) and macroorganisms for terrestrial and marine ecosystems (Hillebrand 2004). The slope of latitudinal diversity in the southern *versus* the northern hemisphere for the epi‐, meso‐ and upper bathypelagic waters was not significantly different (Table S5, Supporting information, ANCOVA, *P* > 0.9) indicating a bipolar distribution of the AOA communities in these water layers (Sul *et al*. 2013). Although we do not have samples collected at strictly ‘polar’ but subpolar locations (64°N–40°N, 55°S–40°S), the trend observed corresponds to what is usually termed ‘bipolar’ distribution (i.e. high latitudes present similar communities and differ from low latitudinal communities). Additional support for the bipolar distribution is provided by the higher amount and percentage of shared OTUs between regions with similar environmental conditions, that is within high and within low latitude regions, than between high and low latitude regions (Fig. 2, Table S3, Supporting information).

**Figure 7:**
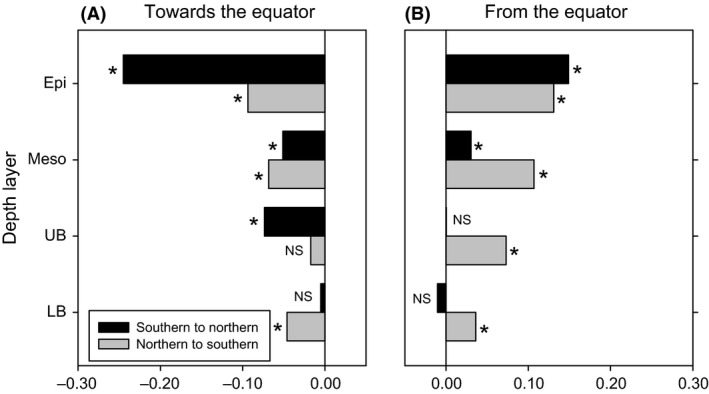
Slope of the decay of similarity with distance from the southernmost AOA community towards the north (black bars) and from the northernmost (grey bars) towards the south, until the Equator (A) and from the Equator on (B). Slope values are in units of ln(similarity) per 1000 km distance. NS, nonsignificant; *significant (*P* < 0.05).

The bipolar distribution of AOA extending from the epi‐ to the upper bathypelagic realm is further supported by the latitudinal variation in beta‐diversity (Fig. 3). This variation results from a decrease in similarity between communities from polar to tropical regions (Fig. 7A) followed by an increase in similarity towards Antarctica or Arctic (Fig. 7B). The decrease in similarity from polar AOA communities towards the equator mimics the distance–decay patterns of macroorganisms. A weaker (or insignificant) slope of the distance–decay in micro‐organisms as compared to macroorganisms is probably attributable to the differences in dispersal limitations between micro‐ and macroorganisms (Soininen *et al*. 2007; Soininen 2012). Indeed, the few studies comparing the patterns of both micro‐ and macroorganisms at the same location support this hypothesis (Mazaris *et al*. 2010; Soininen *et al*. 2011). Within micro‐organisms, eukaryotes exhibit stronger decay patterns than Bacteria (Soininen *et al*. 2011). Although we cannot directly compare our results with those of macroorganisms as we only evaluated AOA in this study, it is still noteworthy that AOA in the Atlantic showed a similar slope in the distance–decay pattern in epipelagic waters as that found for trees, shrubs and bryophytes (Nekola & White 1999), but a steeper slope than that for Bacteria in the International Census of Marine Microbes (ICoMM) study (Zinger *et al*. 2014). However, comparable to Zinger *et al*. (2014), AOA also show a steeper slope in the distance–decay relationship in surface waters than in the deep waters. Few studies compared the distance–decay relationship for Bacteria and Archaea in the same samples (Barreto *et al*. 2014). In the latter study, the methanogenic Archaea targeted by the functional gene *mcr*A showed the steeper slope, followed by the 16S rRNA signature of Archaea and Bacteria (based on 16 rRNA gene abundance), in agreement with our results. This finding might indicate a lower dispersal of AOA as compared to Bacteria, probably explainable by their lower diversity, abundance and growth rate.

The bipolar distribution of AOA supports the Baas‐Becking principle (Baas‐Becking 1934) ‘everything is everywhere, but the environment selects’ and the species‐sorting concept of metacommunity theory (Leibold *et al*. 2004) with weak, albeit stronger, dispersal limits for AOA than for Bacteria. This conclusion is also in agreement with recent findings suggesting the presence of a persistent microbial seed bank in the ocean (Gibbons *et al*. 2013).

The distribution of AOA throughout the Atlantic might also be explained by the Snowball Earth hypothesis (Ashkenazy *et al*. 2013). The Snowball Earth hypothesis (Hoffman & Schrag 2002) is based on the evidence that marine ice extended to the equator during the Neoproterozoic era (~750–635 million years ago) (Ashkenazy *et al*. 2013). In this framework, Ashkenazy *et al*. (2013) suggest that the ocean would have been well‐mixed and was characterized by a dynamic circulation. These dynamic conditions might have resulted in an either well‐mixed or only weakly stratified water column (Ashkenazy *et al*. 2013). Under these conditions, a uniform distribution of microbes can be expected. The following re‐treat of the sea ice and the increasing temperatures in the tropical and temperate ocean might have led to a diversification of the organisms present in these areas, while the high latitudinal organisms would remain relatively unaltered. This hypothesis might also explain the larger latitudinal ranges of high latitude microbes, the higher diversity in low latitude microbes and the higher similarity between Arctic and Southern Ocean polar and subpolar communities.

454‐pyrosequencing and T‐RLP fingerprinting of AOA communities resulted in similar latitudinal and depth‐related patterns, even though these two methods have different resolution level. Similar patterns such as the bipolar distribution, the Rapoport rule or the decrease of beta‐diversity with distance from both northern and southern microbial communities towards the equator were found with both techniques.

In contrast to the epipelagic to upper bathypelagic AOA communities, the AOA communities of the lower bathypelagic (>2000 m) do not show a clear bipolar distribution pattern (Figs 3, 4 and 5, Table S5, Supporting information). The lack of a bipolar distribution pattern in the lower bathypelagic AOA communities might be due the generally small population size restricting dispersal as compared to more abundant populations (Martiny *et al*. 2006). Another possible explanation for the absence of a biogeographic distribution pattern in lower bathypelagic AOA communities might be the influence of resuspension from the sediment on the deepest water masses (Lampitt 1985; Bogucki *et al*. 1997), the presence of a nepheloid layer [i.e. the turbidity layer above the seafloor containing significant amounts of suspended sediment and/or sediment particles (Biscaye & Eittreim 1977)], or the influence of hydrothermal vent plumes (Lupton *et al*. 1985). All these would induce changes in the environmental conditions favouring specific AOA OTUs and resulting in a lower similarity of the communities in this depth layer (Fig. 3) as compared to upper bathypelagic communities. The apparent discrepancy between the lower similarity and the relatively high percentage of shared OTUs (Fig. 2) between lower bathypelagic communities from different regions might be explained by the lower abundance of the shared OTUs. In the lower bathypelagic communities, 69% of the shared OTUs had more than one sequence per sample as compared to 70% and 79% in the upper bathypelagic and mesopelagic communities, respectively. The more variable conditions in near‐bottom waters are also reflected by higher prokaryotic abundance or heterotrophic production (De Corte *et al*. 2012) (see representative profiles for the different regions in Fig. S8, Supporting information) than in the overlying waters.

However, hot spots for dispersal of the AOA community in the bathypelagic Atlantic exist as well. The higher amount and percentage of shared OTUs between depth layers (Fig. 5D) in regions characterized by deep‐water mass formation (ARCT and SANT) or upwelling (WTRA) indicate that these regions might act as hot spots for dispersion of micro‐organisms in the bathypelagic realm. The notion of higher dispersal of micro‐organisms in these areas is also supported by the higher diversity of the lower bathypelagic AOA communities (Fig. 1) in the ARCT and SANT as compared to the NADR and gyral provinces.

Taken together, our results indicate that epipelagic and deep‐water AOA follow biogeographic distribution patterns, similar to Bacteria and macroorganisms, though with some differences in the shape of the relationships. More specifically, AOA exhibit a bipolar distribution of AOA communities from epi‐ to upper bathypelagic waters (<2000 m). The bipolar distribution of surface and deep ocean AOA might be interpreted in the frame of the Baas‐Becking principle and the species‐sorting theory, or as a result of an historical homogeneous period (Snowball Earth hypothesis) followed by changing environmental conditions that favoured speciation and diversification in low latitudes. Moreover, distinct hot spots for archaeal dispersal have been identified, such as the formation sites of deep‐water masses and upwelling zones, pointing to the importance of these areas to sustain the diversity of the deep ocean microbial communities. The contrasting biogeographic pattern of the AOA communities inhabiting the deepest layers (>2000 m) might be a consequence of sporadic events affecting near‐bottom water layers and introducing variability in the environmental conditions, as well as by their small population size.

E.S. and G.J.H. designed the work and wrote the study. E.S., D.D.C. and N.O. performed research. E.S. performed all the data analysis.

## Data accessibility

Sequence information obtained by Sanger sequencing has been deposited in GenBank, Accession nos KF727022‐KF727275. Raw 454‐pyrosequences were submitted to the Sequence Read Archive (SRA) at NCBI under the Accession number SRP049002. Station GPS coordinates and sampling date, OTUs abundance obtained by T‐RFLP and 454‐pyrosequencing have been deposited on Dryad (doi:10.5061/dryad.c6688).

## Supporting information


**Fig. S1** Location of the sampling stations along the cruise track in the Atlantic Ocean.
**Fig. S2** Rarefaction curves at a 98% identity cutoff for OTU assignment showing the relative richness of the *amo*A gene in different regions and depth layers of the Atlantic: (A) obtained from cloning and sequencing, (B) obtained from 454‐forward pyrosequenced libraries, (C) obtained from 454‐reverse pyrosequenced libraries.
**Fig. S3** MDS ordination of similarity (Bray‐Curtis) for the archaeal ammonia oxidizer community: from (A) epipelagic, (B) mesopelagic, (C) upper bathypelagic and (D) lower bathypelagic waters throughout the Atlantic obtained by T‐RFLP fingerprinting.
**Fig. S4** Shared OTUs between depth layers.
**Fig. S5** Relationship between AOA average temperature range and temperature. Each symbol represents the average temperature range for a sample based on the TRFLP OTUs present at different depth layers: Epi, epipelagic, Meso, mesopelagic, UB, upper bathypelagic, LB, lower bathypelagic.
**Fig. S6** Canonical correspondence analysis: for the (A) T‐RFLP fingerprints from the reverse region, and (B) 454‐pyrosequencing libraries sequenced with the reverse primer.
**Fig. S7** Venn diagram showing the contribution of environmental, spatial and temporal factors to the explained variation of the AOA community composition in the Atlantic ocean.
**Fig. S8** Depth profiles of one representative station from each oceanographic region in the Atlantic (named as in Fig. S1): of (A) prokaryotic abundance and (B) heterotrophic production. Prokaryotic abundance and production of the entire northern transect is given in De Corte *et al*. (2012).

**Table S1** Stations and depths where samples for cloning and/or 454‐pyrosequencing of archaeal *amo*A were collected during the Geotraces cruises.
**Table S2** Chao richness index (Chao), ACE richness index (Ace), Shannon diversity index (H') and Simpson diversity index (S) of OTUs (defined at 98% similarity) obtained from the AOA clone libraries (cloning) and from the 454 forward (454‐F) and reverse (454‐R) pyro‐sequenced libraries throughout the Atlantic.
**Table S3** Number of shared OTUs (98% similarity) between regions for the whole water column and for specific depths for the forward and reverse 454 pyro‐sequenced *amo*A gene.
**Table S4** Mean latitudinal range for low abundant AOA OTUs (>0.5% of the total peak height in T‐RFLP fingerprints, LA) vs. high abundant OTUs (>5% of the total peak height, HA) and *vs*. the 454‐pyrosequenced OTUs (454) obtained for different depth layers.
**Table S5** Adjusted mean and slope of the variation of the Shannon index of diversity *versus* latitude in the northern (N) and southern (S) hemispheres obtained with T‐RFLP and 454‐pyrosequencing (454‐pyro).

**Appendix S1** Material and methods.
