# Supplementary material for: Macroecological patterns of archaeal ammonia oxidizers in the Atlantic Ocean
Source: Mol Ecol. 2015 Sep 28;24(19):4931–42. doi: 10.1111/mec.13365 (PMC4950044; doi:10.1111/mec.13365)
Supplement: Supplementary file 1 — Fig. S1 Location of the sampling stations along the cruise track in the Atlantic Ocean. Fig. S2 Rarefaction curves at a 98% identity cutoff for OTU assignment showing the relative richness of the amoA gene in different regions and depth layers of the Atlantic: (A) obtained from cloning and sequencing, (B) obtained from 454‐forward pyrosequenced libraries, (C) obtained from 454‐reverse pyrosequenced libraries. Fig. S3 MDS ordination of similarity (Bray‐Curtis) for the archaeal ammonia oxidizer community: from (A) epipelagic, (B) mesopelagic, (C) upper bathypelagic and (D) lower bathypelagic waters throughout the Atlantic obtained by T‐RFLP fingerprinting. Fig. S4 Shared OTUs between depth layers. Fig. S5 Relationship between AOA average temperature range and temperature. Each symbol represents the average temperature range for a sample based on the TRFLP OTUs present at different depth layers: Epi, epipelagic, Meso, mesopelagic, UB, upper bathypelagic, LB, lower bathypelagic. Fig. S6 Canonical correspondence analysis: for the (A) T‐RFLP fingerprints from the reverse region, and (B) 454‐pyrosequencing libraries sequenced with the reverse primer. Fig. S7 Venn diagram showing the contribution of environmental, spatial and temporal factors to the explained variation of the AOA community composition in the Atlantic ocean. Fig. S8 Depth profiles of one representative station from each oceanographic region in the Atlantic (named as in Fig. S1): of (A) prokaryotic abundance and (B) heterotrophic production. Prokaryotic abundance and production of the entire northern transect is given in De Corte et al. (2012). [file MEC-24-4931-s001.pdf]

SUPPLEMENTARY FIGURE LEGENDS

Figure S1. Location of the sampling stations along the cruise track in the Atlantic Ocean. Oceanographic regions based on Longhurst (Longhurst 2007) are indicated by thick lines. ARCT: North Atlantic Arctic Province, NADR: North Atlantic Drift Province, NAG: North Atlantic Gyral Province, WTRA: Western Tropical Atlantic, SATL: South Atlantic Gyral Province, SANT: Subantarctic Province. Stations where 454-pyrosequencing of the *amo*A gene was conducted are marked with an arrow.

Figure S2. Rarefaction curves at a 98% identity cutoff for OTU assignment showing the relative richness of the *amo*A gene in different regions and depth layers of the Atlantic: (A) obtained from cloning and sequencing, (B) obtained from 454-forward pyrosequenced libraries, (C) obtained from 454-reverse pyrosequenced libraries.

Figure S3. MDS ordination of similarity (Bray-Curtis) for the archaeal ammonia oxidizer community: from (A) epipelagic, (B) mesopelagic, (C) upper bathypelagic and (D) lower bathypelagic waters throughout the Atlantic obtained by T-RFLP fingerprinting. Symbols represent different oceanographic regions, named as in Fig. S1. Lines engulfing samples represent the specific similarity value.

Figure S4. Shared OTUs between depth layers. Venn diagram showing the shared OTUs (98%) between the mesopelagic (250 m, light blue), upper bathypelagic (1250 m, blue) and lower bathypelagic (2500-4500 m, dark blue) AOA communities in different oceanographic regions of the Atlantic (named as in Fig. 1), obtained with forward sequencing (A) or reverse sequencing (B). Salinity measured along the transect is indicated as a reference (C), arrows indicate the stations sampled for 454-pyrosequencing. Percentage of shared OTUs throughout the water column in different regions (D).

Figure S5. Relationship between AOA average temperature range and temperature. Each symbol represents the average temperature range for a sample based on the T-RFLP OTUs present at different depth layers: Epi, epipelagic, Meso, mesopelagic, UB, upper bathypelagic, LB, lower bathypelagic.

Figure S6. Canonical correspondence analysis: for the (A) T-RFLP fingerprints from the reverse region, and (B) 454-pyrosequencing libraries sequenced with the reverse primer. Symbols denote different provinces, named as in Fig. S1. Arrows indicate selected environmental variable (Latitude, Depth, Sal: salinity, Temp: temperature, oxy: dissolved oxygen concentration, NO2: nitrite concentration, MCGI/mL SW: 16S rRNA gene abundance of Thaumarchaeota, LAC/HACamoA: ratio between LAC and HAC-*amo*A gene). Sample depth of the individual 454-pyrosequencing libraries is indicated next to the symbols.

Figure S7. Venn diagram showing the contribution of environmental, spatial and temporal factors to the explained variation of the AOA community composition in the Atlantic ocean.

Figure S8. Depth profiles of one representative station from each oceanographic region in the Atlantic (named as in Fig. S1): of (A) prokaryotic abundance and (B)

heterotrophic production. Prokaryotic abundance and production of the entire northern transect is given in De Corte et al (2012).

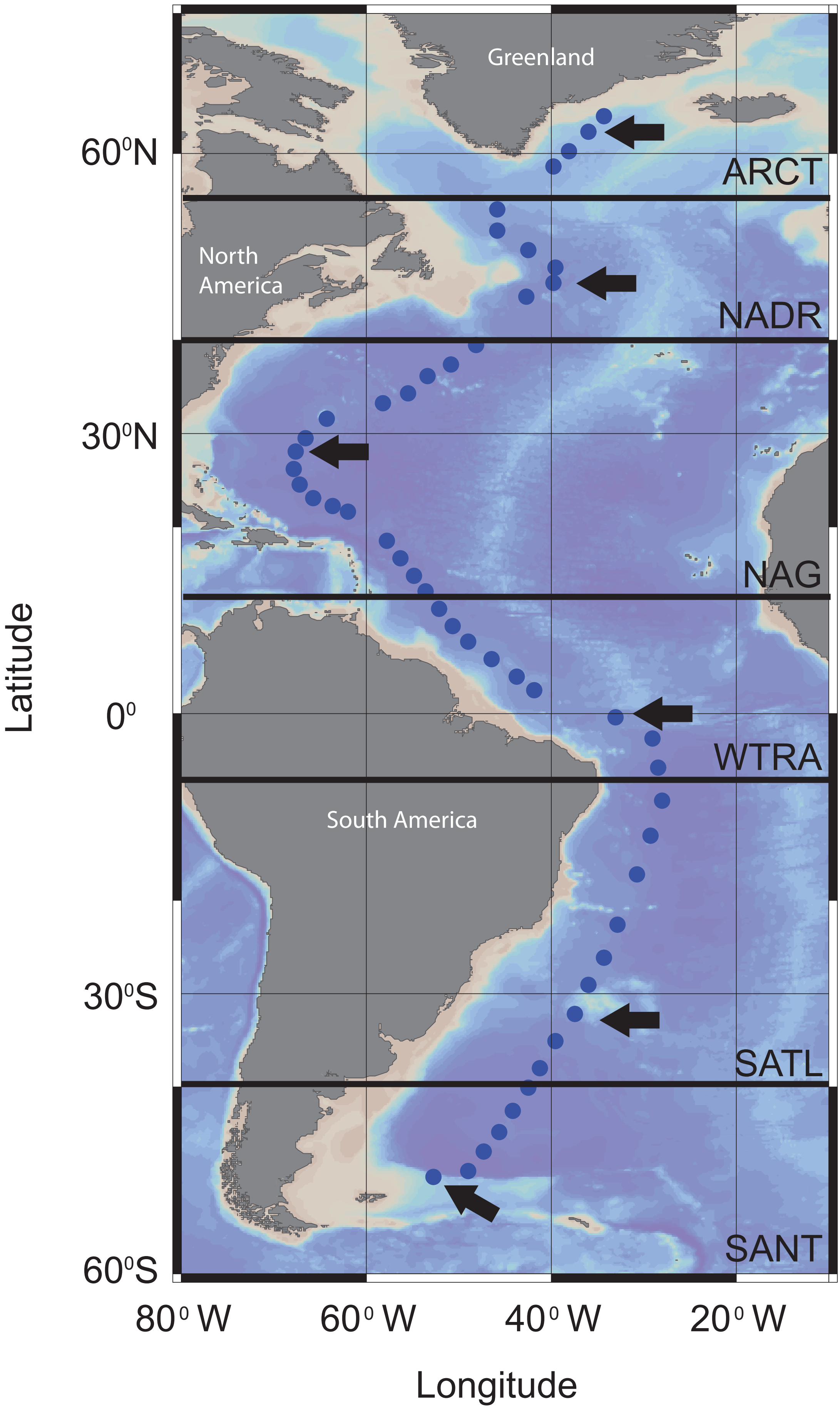

Figure S1. Sintes et al.

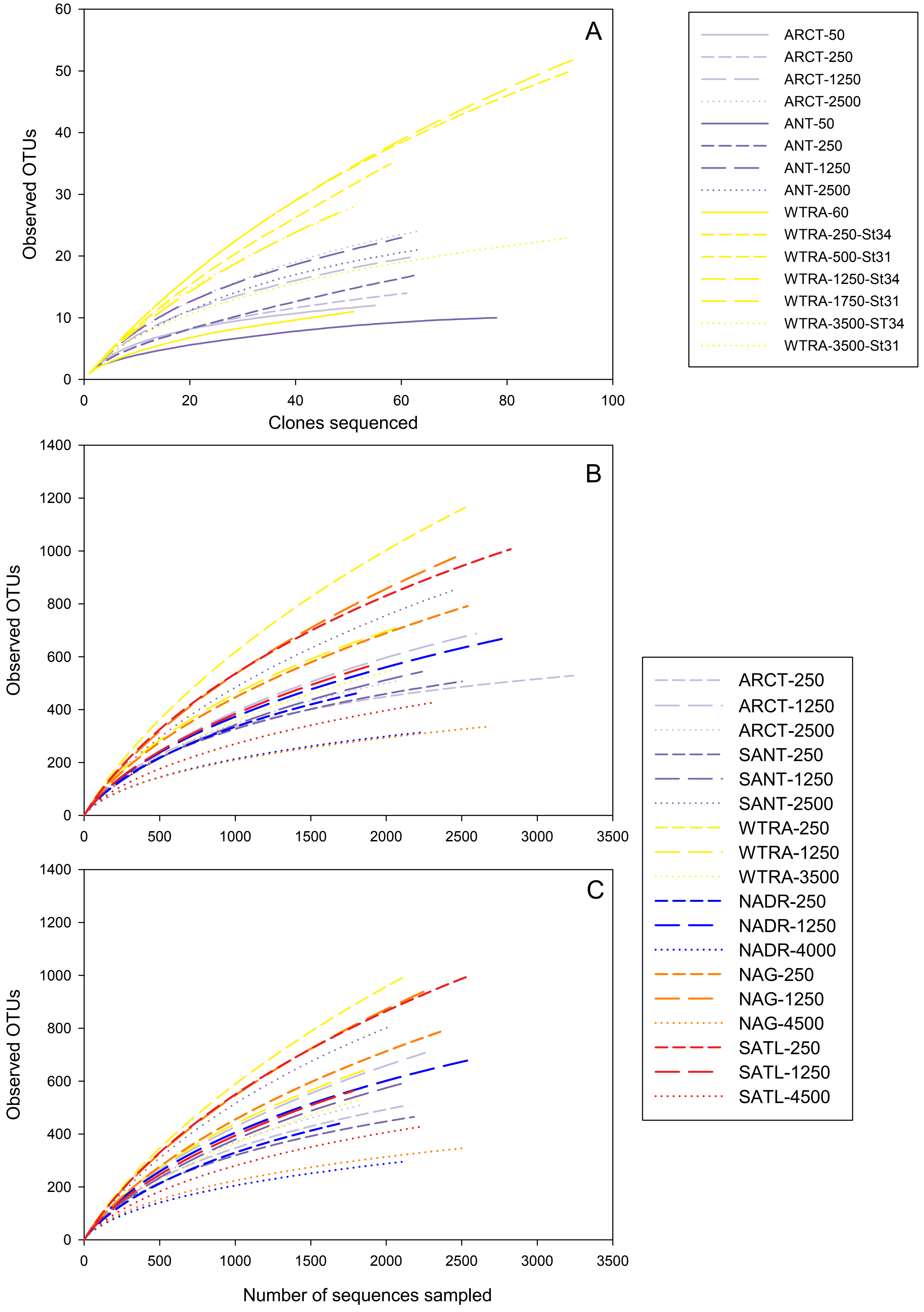

Fig. S2. Sintes et al.

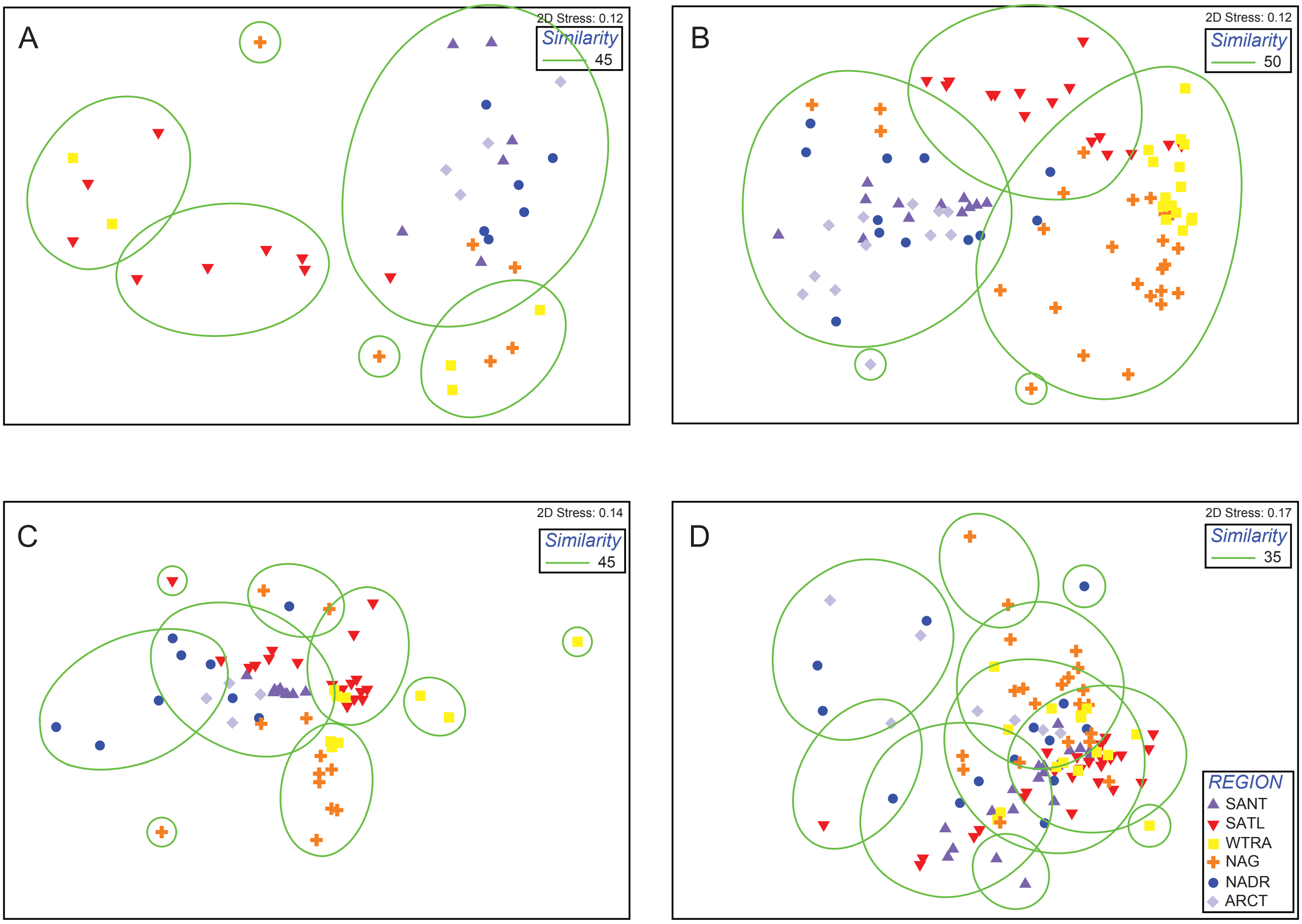

Figure S3. Sintes et al.

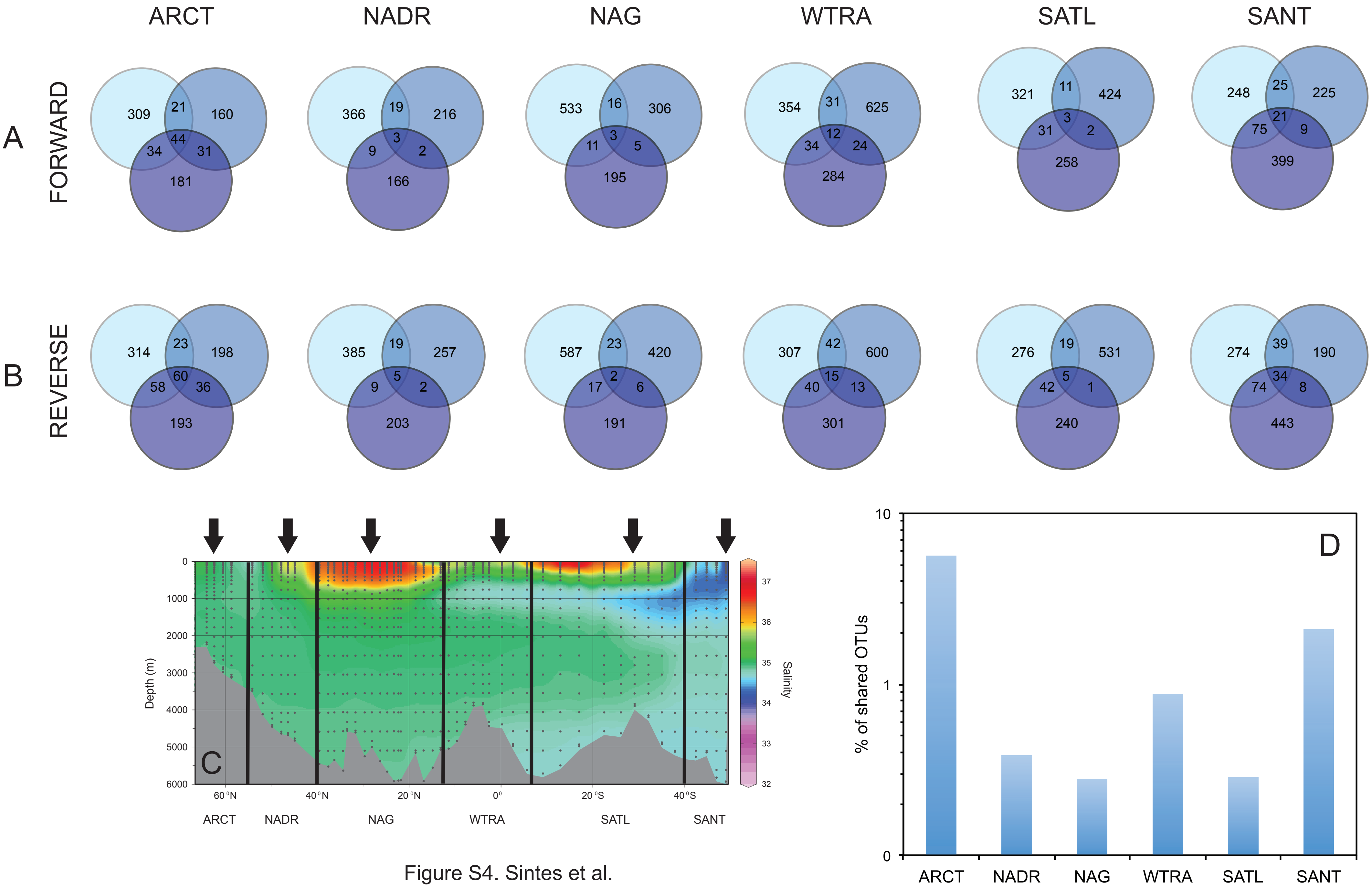

Figure S4. Sintes et al.

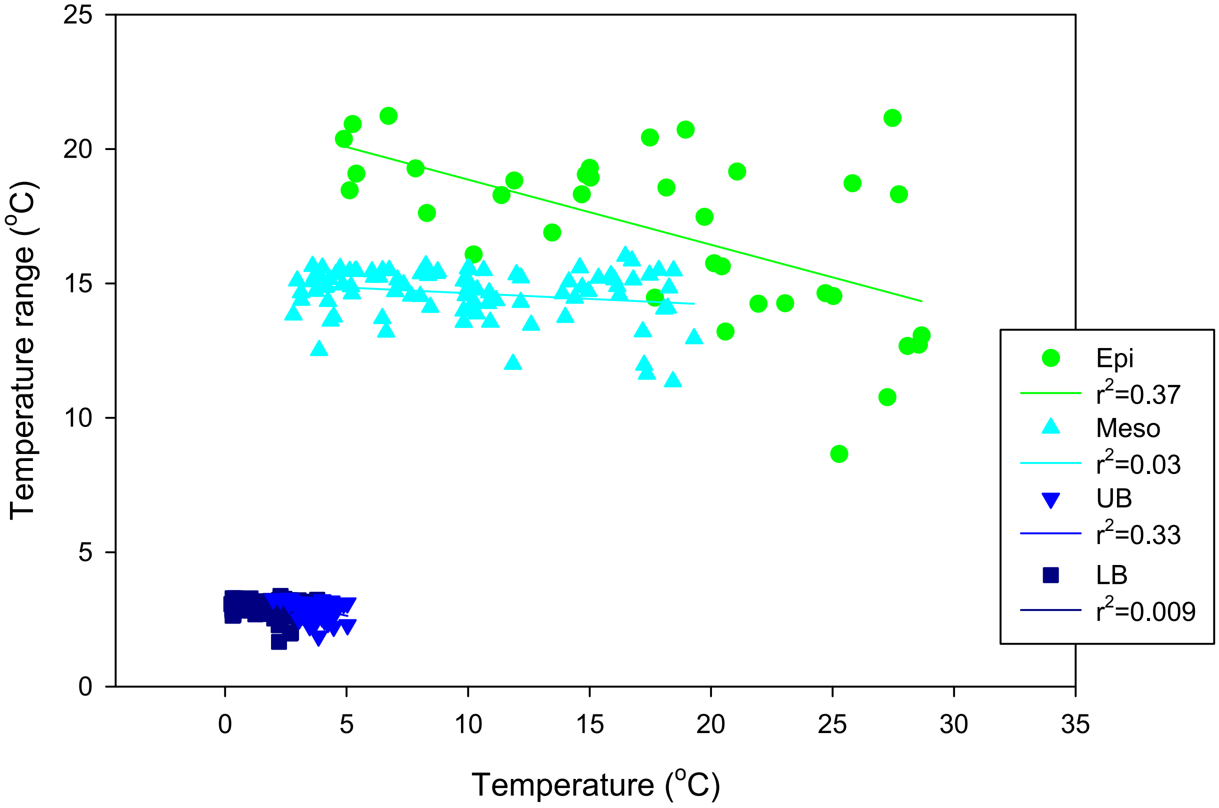

Figure S5. Sintes et al.

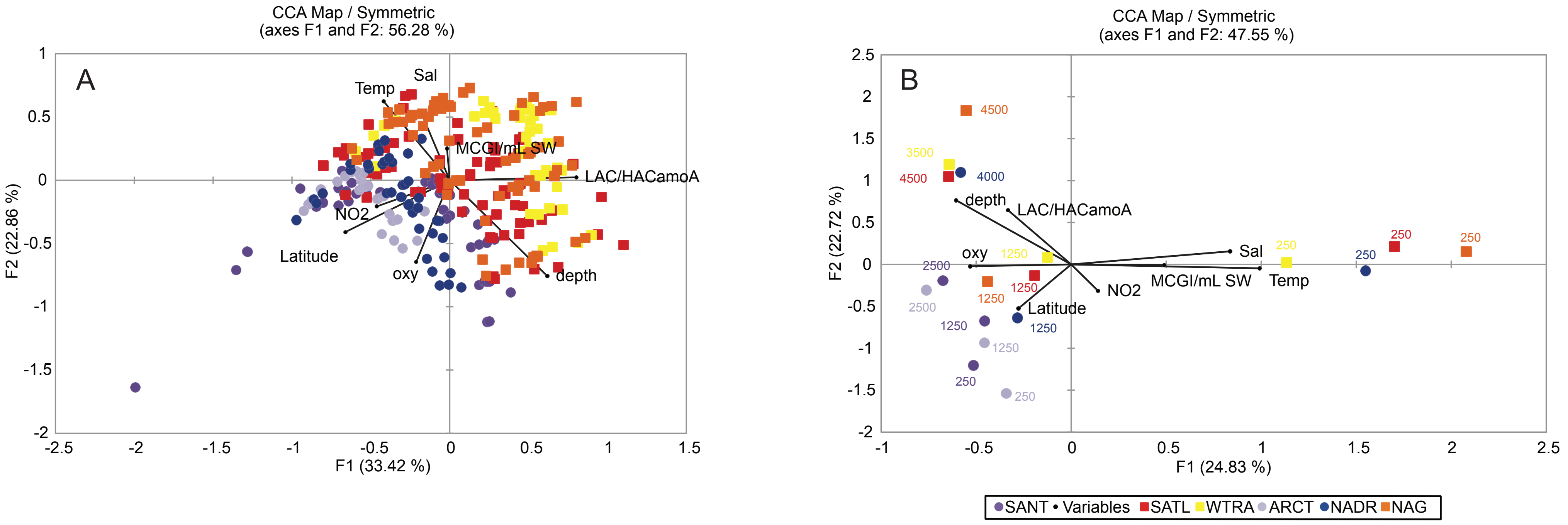

Figure S6. Sintes et al.

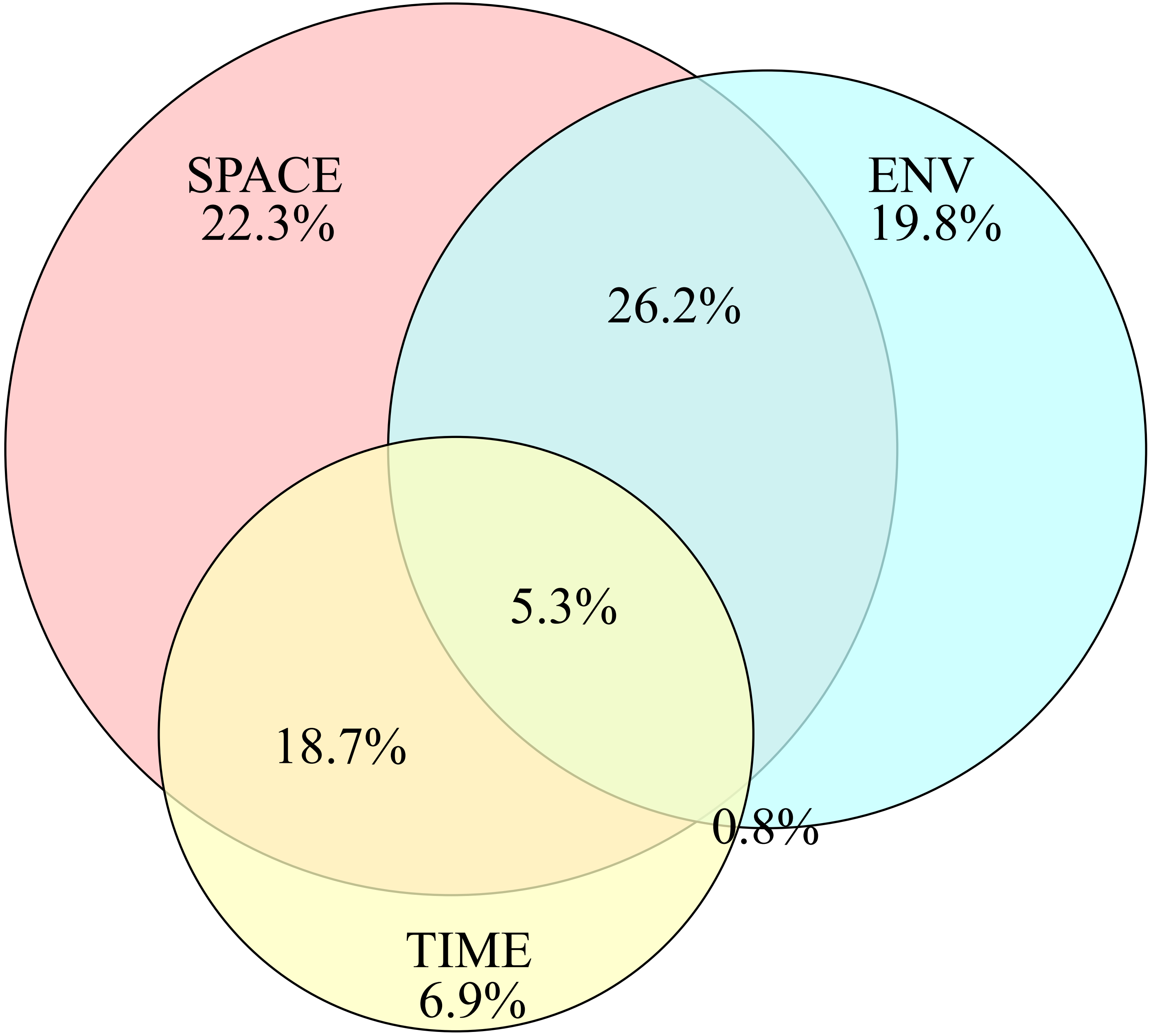

Figure S7. Sintes et al.

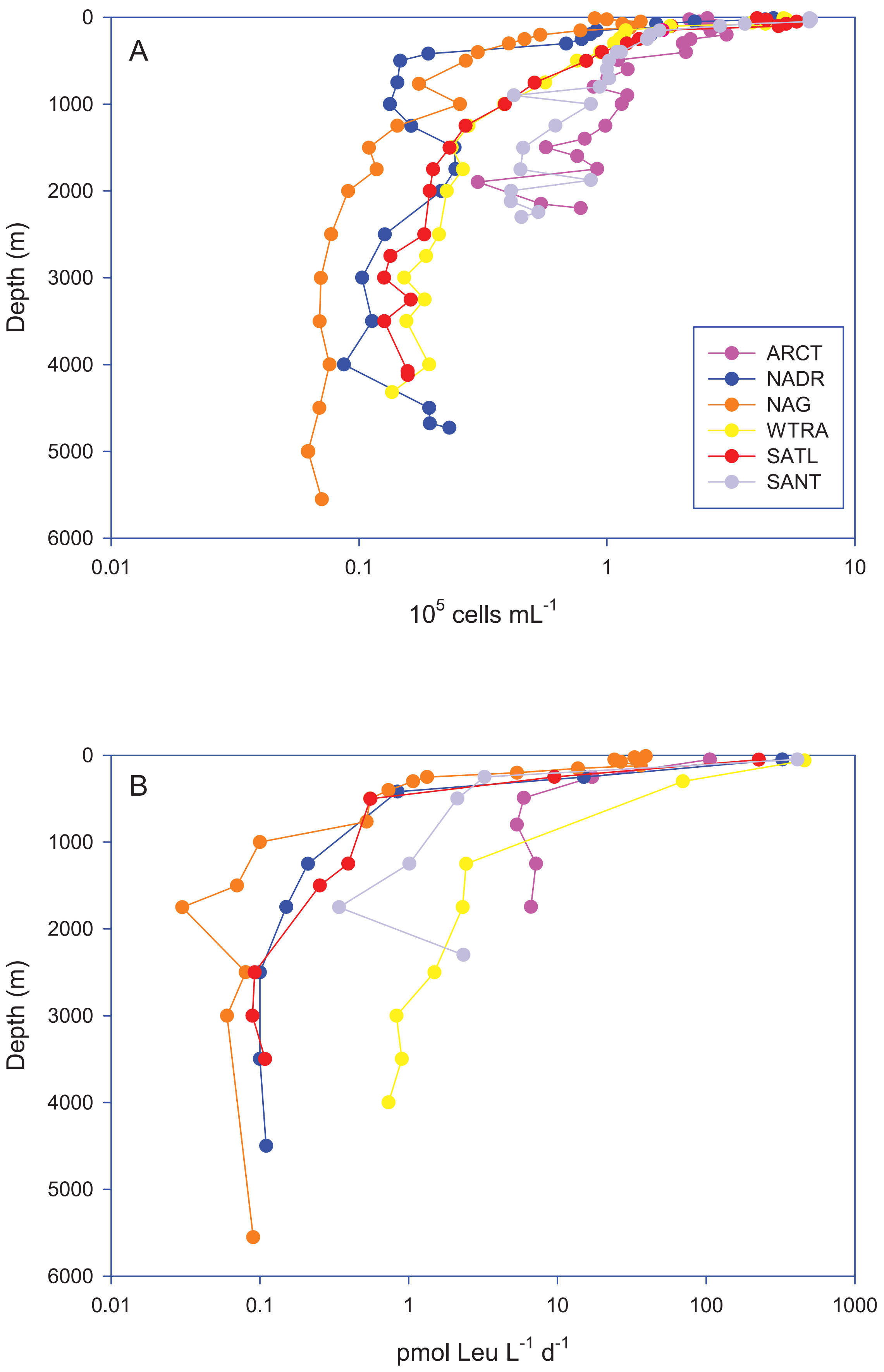

Figure S8. Sintes et al.
