# Supplementary material for: Macroecological patterns of archaeal ammonia oxidizers in the Atlantic Ocean
Source: Mol Ecol. 2015 Sep 28;24(19):4931–42. doi: 10.1111/mec.13365 (PMC4950044; doi:10.1111/mec.13365)
Supplement: Supplementary file 2 — Table S1 Stations and depths where samples for cloning and/or 454‐pyrosequencing of archaeal amoA were collected during the Geotraces cruises. Table S2 Chao richness index (Chao), ACE richness index (Ace), Shannon diversity index (H') and Simpson diversity index (S) of OTUs (defined at 98% similarity) obtained from the AOA clone libraries (cloning) and from the 454 forward (454‐F) and reverse (454‐R) pyro‐sequenced libraries throughout the Atlantic. Table S3 Number of shared OTUs (98% similarity) between regions for the whole water column and for specific depths for the forward and reverse 454 pyro‐sequenced amoA gene. Table S4 Mean latitudinal range for low abundant AOA OTUs (>0.5% of the total peak height in T‐RFLP fingerprints, LA) vs. high abundant OTUs (>5% of the total peak height, HA) and vs. the 454‐pyrosequenced OTUs (454) obtained for different depth layers. Table S5 Adjusted mean and slope of the variation of the Shannon index of diversity versus latitude in the northern (N) and southern (S) hemispheres obtained with T‐RFLP and 454‐pyrosequencing (454‐pyro). [file MEC-24-4931-s002.doc]

**Table S1.** Stations and depths where samples for cloning and/or 454-pyrosequencing of archaeal *amo*A were collected during the Geotraces cruises.

| Station | Depth | Analysis | Geographic area |
| --- | --- | --- | --- |
| 2 | 50 | Cloning | ARCT |
|  | 250, 1250, 2500 | Cloning, 454-pyro |  |
| 9 | 250, 1250, 4000 | 454-pyro | NADR |
| 18 | 250, 1250, 4500 | 454-pyro | NAG |
| 31 | 500, 1750, 3500 | Cloning | WTRA |
| 34 | 60 | Cloning | WTRA |
|  | 250, 1250, 3500 | Cloning, 454-pyro |  |
| 43 | 250, 1250, 4500 | 454-pyro | SATL |
| 51 | 50 | Cloning | SANT |
|  | 250, 1250, 2500 | Cloning, 454-pyro |  |

**Table S2.** Chao richness index (Chao), ACE richness index (Ace), Shannon diversity index (H’) and Simpson diversity index (S) of OTUs (defined at 98% similarity) obtained from the AOA clone libraries (cloning) and from the 454 forward (454-F) and reverse (454-R) pyro-sequenced libraries throughout the Atlantic. All the indexes have been calculated based on the lowest common minimum number of clones from all clone libraries. Highest value for each station is marked in bold.

|  |  |  | Cloning | | | | 454-F | | | | 454-R | | | |
| --- | --- | --- | --- | --- | --- | --- | --- | --- | --- | --- | --- | --- | --- | --- |
| Region | Station | Depth (m) | Ace | H’ | S | Chao | Ace | H’ | S | Chao | Ace | H’ | S | Chao |
| ARCT | St2 | 2500 | 32 | 2.72 | 0.066 | 25 | 1086 | 5.31 | 0.014 | 851 | 1075 | 5.47 | **0.009** | 826 |
|  |  | 1250 | **50** | **2.83** | **0.064** | **36** | **1566** | 5.41 | 0.016 | **1131** | **2042** | **5.68** | **0.009** | **1325** |
|  |  | 250 | 17 | 1.93 | 0.209 | 15 | 663 | **5.45** | **0.010** | 700 | 1038 | 5.42 | 0.010 | 795 |
|  |  | 50 | 9 | 1.76 | 0.186 | 9 | - | - | - | - | - | - | - | - |
| NADR | St9 | 4000 | - | - | - | - | 488 | 4.25 | 0.049 | 471 | 625 | 4.17 | 0.053 | 470 |
|  |  | 1250 | - | - | - | - | **1661** | **5.43** | **0.015** | **1157** | **1380** | **5.61** | **0.012** | **1050** |
|  |  | 250 | - | - | - | - | 802 | 4.17 | 0.026 | 768 | 774 | 4.91 | 0.034 | 765 |
| NAG | St18 | 4500 | - | - | - | - | 674 | 4.42 | 0.032 | 566 | 656 | 4.41 | 0.038 | 549 |
|  |  | 1250 | - | - | - | - | **3056** | **6.17** | **0.005** | **2082** | **2911** | **6.22** | **0.004** | **1930** |
|  |  | 250 | - | - | - | - | 2117 | 5.80 | 0.008 | 1451 | 2143 | 5.71 | 0.014 | 1580 |
| WTRA | St31 | 3500 | 25 | 2.32 | 0.111 | 22 | - | - | - | - | - | - | - | - |
|  |  | 1750 | **125** | 3.08 | 0.044 | 113 | - | - | - | - | - | - | - | - |
|  |  | 500 | 107 | **3.34** | **0.021** | **141** | - | - | - | - | - | - | - | - |
| WTRA | St34 | 3500 | 59 | **3.09** | **0.035** | **78** | 1377 | 5.05 | 0.026 | 1011 | 1417 | 4.98 | 0.033 | 1002 |
|  |  | 1250 | 52 | 2.92 | 0.065 | 45 | 1732 | 5.81 | 0.009 | 1206 | 1815 | 5.70 | 0.008 | 1214 |
|  |  | 250 | **78** | 3.03 | 0.044 | 70 | **3637** | **6.58** | **0.002** | **2436** | **3452** | **6.39** | **0.003** | **2142** |
|  |  | 50 | 22 | 1.51 | 0.368 | 16 | - | - | - | - | - | - | - | - |
| SATL | St43 | 4500 | - | - | - | - | 955 | 4.70 | 0.027 | 654 | 703 | 4.64 | 0.037 | 648 |
|  |  | 1250 | - | - | - | - | 1730 | 5.36 | 0.017 | 1171 | 1643 | 5.41 | 0.012 | 1092 |
|  |  | 250 | - | - | - | - | **2261** | **6.27** | **0.004** | **1665** | **2617** | **6.25** | **0.006** | **1786** |
| SANT | St51 | 2500 | **23** | 2.48 | 0.117 | 21 | **2271** | **5.93** | **0.008** | **1591** | **1548** | **6.00** | **0.006** | **1367** |
|  |  | 1250 | 22 | **2.52** | **0.076** | **27** | 1213 | 5.13 | 0.018 | 938 | 1419 | 5.21 | 0.022 | 1133 |
|  |  | 250 | 18 | 1.70 | 0.235 | 15 | 776 | 5.29 | 0.013 | 724 | 833 | 5.20 | 0.015 | 658 |
|  |  | 50 | 4 | 1.20 | 0.321 | 4 | - | - | - | - | - | - | - | - |

**Table S3.** Number of shared OTUs (98% similarity) between regions for the whole water column and for specific depths for the forward and reverse 454 pyro-sequenced *amo*A gene. Highest number of shared OTUs for every region is marked in bold.

| **Forward** |  | NADR | NAG | WTRA | SATL | SANT |
| --- | --- | --- | --- | --- | --- | --- |
| All depths | ARCT | 237 | 166 | 115 | 153 | **238** |
|  | NADR |  | **238** | 165 | 213 | 190 |
|  | NAG |  |  | **331** | **313** | 178 |
|  | WTRA |  |  |  | 312 | 148 |
|  | SATL |  |  |  |  | 196 |
| Meso | ARCT | 37 | 12 | 16 | 28 | **87** |
|  | NADR |  | 62 | 36 | **87** | 13 |
|  | NAG |  |  | 74 | **114** | 4 |
|  | WTRA |  |  |  | **109** | 12 |
|  | SATL |  |  |  |  | 11 |
| UpperBathy | ARCT | **128** | **130** | 63 | 102 | 110 |
|  | NADR |  | 116 | 49 | 73 | 83 |
|  | NAG |  |  | **154** | **135** | **113** |
|  | WTRA |  |  |  | 97 | 63 |
|  | SATL |  |  |  |  | 106 |
| LowerBathy | ARCT | 64 | 54 | 69 | 49 | **103** |
|  | NADR |  | 84 | **94** | 73 | 88 |
|  | NAG |  |  | **116** | 91 | 87 |
|  | WTRA |  |  |  | **114** | 115 |
|  | SATL |  |  |  |  | 104 |
| **Reverse** |  | NADR | NAG | WTRA | SATL | SANT |
| All depths | ARCT | **256** | 157 | 89 | 147 | **285** |
|  | NADR |  | 247 | 156 | 234 | 219 |
|  | NAG |  |  | **349** | **357** | 195 |
|  | WTRA |  |  |  | 327 | 177 |
|  | SATL |  |  |  |  | 209 |
| Meso | ARCT | 41 | 8 | 9 | 20 | **88** |
|  | NADR |  | 81 | 32 | **104** | 18 |
|  | NAG |  |  | 82 | 144 | 4 |
|  | WTRA |  |  |  | 116 | 10 |
|  | SATL |  |  |  |  | 9 |
| UpperBathy | ARCT | **143** | 124 | 41 | 100 | **135** |
|  | NADR |  | 93 | 32 | 61 | 91 |
|  | NAG |  |  | 133 | **140** | 125 |
|  | WTRA |  |  |  | 109 | 68 |
|  | SATL |  |  |  |  | 114 |
| LowerBathy | ARCT | 38 | 16 | 24 | 23 | **69** |
|  | NADR |  | 62 | **73** | 61 | 71 |
|  | NAG |  |  | **91** | 70 | 45 |
|  | WTRA |  |  |  | **119** | 85 |
|  | SATL |  |  |  |  | **91** |

**Table S4.** Mean latitudinal range for low abundant AOA OTUs (>0.5% of the total peak height in T-RFLP fingerprints, LA) vs. high abundant OTUs (>5% of the total peak height, HA) and *vs*. the 454-pyrosequenced OTUs (454) obtained for different depth layers. P <0.05 indicates significant difference between the mean values (paired t-test). Epi: epipelagic, Meso: Mesopelagic, UB: Upper bathypelagic, LB: lower bathypelagic.

|  | | North  (n=18-23) | | South  (n=18) | | All  (n=36-41) | | All (454 *vs*. T-RFLP, n=6) | |
| --- | --- | --- | --- | --- | --- | --- | --- | --- | --- |
|  |  | Mean | P | Mean | P | Mean | P | Mean | P |
| Epi | LA | 84.60 | **0.01** | 83.61 | 0.18 | 84.11 | **0.006** |  |  |
|  | HA | 89.34 |  | 86.41 |  | 87.87 |  |  |  |
| Meso | 454 |  |  |  |  |  |  | 38.19 |  |
|  | LA | 92.08 | **<0.001** | 101.90 | 0.72 | 95.08 | **0.003** | 96.26 | **0.004** |
|  | HA | 99.03 |  | 102.31 |  | 98.05 |  | 97.50 | **0.003** |
| UB | 454 |  |  |  |  |  |  | 32.12 |  |
|  | LA | 89.99 | **0.006** | 100.58 | **<0.001** | 94.75 | **<0.001** | 93.54 | **<0.001** |
|  | HA | 95.34 |  | 105.58 |  | 100.0 |  | 98.54 | **<0.001** |
| LB | 454 |  |  |  |  |  |  | 28.64 |  |
|  | LA | 97.04 | 0.45 | 96.87 | **<0.001** | 96.96 | **<0.001** | 95.82 | **<0.001** |
|  | HA | 98.01 |  | 103.15 |  | 100.27 |  | 96.85 | **<0.001** |

**Table S5.** Adjusted mean and slope of the variation of the Shannon index of diversity *versus* latitude in the northern (N) and southern (S) hemispheres obtained with T-RFLP and 454-pyrosequencing (454-pyro). P value for equality of adjusted means and slope of northern versus southern variation is indicated (ANCOVA analysis). P<0.05 indicates significant difference between means or slopes (marked in bold).

|  |  | T-RFLP | | | | 454-pyro | | | |
| --- | --- | --- | --- | --- | --- | --- | --- | --- | --- |
|  |  | Ad. Mean | Slope | Equal ad. means | Equal slope | Ad. Mean | Slope | Equal ad. means | Equal slope |
| Epi | N | 2.889 | -0.002 | 0.54 | 0.996 | - | - | - | - |
|  | S | 2.994 | -0.002 |  |  | - | - |  |  |
| Meso | N | 3.324 | -0.001 | 0.95 | 0.96 | 5.325 | -0.012 | 0.49 | 0.81 |
|  | S | 3.328 | -0.001 |  |  | 5.861 | -0.024 |  |  |
| UpperBathy | N | 3.248 | 0.006 | 0.85 | 0.93 | 5.822 | -0.022 | **0.05** | 0.46 |
|  | S | 3.259 | 0.006 |  |  | 5.281 | -0.014 |  |  |
| LowerBathy | N | 3.061 | -0.005 | 0.88 | **0.04** | 4.497 | 0.025 | 0.17 | 0.77 |
|  | S | 3.052 | 0.003 |  |  | 5.389 | 0.014 |  |  |
