# Supplementary material for: Macroecological patterns of archaeal ammonia oxidizers in the Atlantic Ocean
Source: Mol Ecol. 2015 Sep 28;24(19):4931–42. doi: 10.1111/mec.13365 (PMC4950044; doi:10.1111/mec.13365)
Supplement: Supplementary file 3 — Appendix S1 Material and methods. [file MEC-24-4931-s003.docx]

**SUPPLEMENTARY TEXT S1**

**MATERIAL AND METHODS**

**Inorganic nutrient concentrations**

The concentrations of dissolved inorganic nutrients (NO_3_^-^, NO_2_^-^, PO_4_^3-^) were determined on 0.2 µm filtered water samples in a TRAACS 800 autoanalyzer system ([Reinthaler *et al.* 2008](#_ENREF_11)).

**Abundance of the microbial community**

Two mL samples were fixed with glutaraldehyde (0.5% final concentration), flash-frozen in liquid N2 and kept at -80ºC until analysis. To enumerate prokaryotes by flow cytometry, samples were thawed to room temperature and 0.5 mL subsamples stained with SYBR Green I in the dark for 10 min and subsequently, 1 x 10^5^ mL^-1^ of 1 µm fluorescent polystyrene beads (Molecular Probes, Invitrogen) were added to each sample as internal standard. The prokaryotes were enumerated on a FACSAria II flow cytometer (Becton Dickinson) based on their signature in a plot of green fluorescence versus side scatter as previously described ([De Corte *et al.* 2012](#_ENREF_3)).

**DNA extraction**

Two to 10 L of seawater were filtered through 0.22 µm GTTP polycarbonate filters (Millipore) depending on the depth. Subsequently, the filters were stored at -80ºC until processed in the home laboratory. The extraction was performed using Ultraclean soil DNA isolation kit (Mobio).

**Preparation of the q-PCR standards**

The standards for the 16S rRNA gene of Marine *Crenarchaeota* Group I (MCGI, recently coined Thaumarchaeota) and the two archaeal *amo*A were prepared from the plasmid 88exp4 (from the archaeal clones library), from *Nitrosopumilus maritimus* (obtained from C. Schleper, University of Vienna) and from a deep sea sample as described previously ([Agogué *et al.* 2008](#_ENREF_1); [Sintes *et al.* 2013](#_ENREF_14)) using specific primers. Based on a previous study ([Sintes *et al.* 2013](#_ENREF_14)), two clusters of archaeal *amo*A were distinguished: the ‘low-ammonia concentration’ archaeal *amo*A (LAC-archaeal *amo*A) and the ‘high-ammonia concentration’ archaeal *amo*A (HAC-archaeal *amo*A). The specific primers used were MCGI-391F, 5’ AAGGTTARTCCGAGTGRTTTC and MCGI-554R, 5’ TGACCACTTGAGGTGCTG for 16S rRNA of Thaumarchaeota ([Wuchter *et al.* 2006](#_ENREF_19)); the arch-*amo*A-For, 5’ CTGAYTGGGCYTGGACATC and arch-*amo*A-Rev, 5’ TTCTTCTTTGTTGCCCAGTA for HAC-*amo*A, and the arch-*amo*A-For and arch-*amo*A-Rev-New, 5’ TTCTTCTTCGTCGCCCAATA for LAC-*amo*A (Sintes *et al*., 2013).

Each amplification was performed under the following conditions: 4 min initial denaturation; 35 cycles at 94°C for 30 s, specific annealing temperature of the primer set for 40 s (61ºC for MCGI, 58.5ºC for the two archaeal *amo*A primer combinations), 72°C for 2 min, 80°C for 25 s. The reaction mixture (50 µL) consisted of 1 U of PicoMaxx high fidelity DNA polymerase (Agilent Technologies), 1x PicoMaxx PCR buffer, 0.25 mM of each dNTP, 8 µg of BSA, 0.2 µM of primers, 3mM of MgCl_2_ and ultra pure sterile water (Sigma). Amplification products were checked on an agarose gel (2%) after staining with SYBRGold^®^ (Invitrogen). PCR products were purified using PCRExtract MiniKit (5-PRIME). Purified products were quantified using a Nanodrop^®^ spectrophotometer and the abundance of the 16S rRNA and *amo*A genes were subsequently calculated from the concentration of the purified DNA and the size fragment. Ten-fold serial dilutions ranging from 10^7^ to 10^0^ gene copies of the corresponding standard were used in triplicate per q-PCR reaction to generate an external quantification standard.

**Q-PCR analysis**

Q-PCR analysis was performed at all 51 stations and at 6-8 depths per station. All q-PCR analyses were performed on a LightCycler 480 thermocycler (Roche) equipped with LightCycler 480 gene scanning software (version 1.5, Roche). The gene abundance of MCGI 16S rRNA gene, LAC-archaeal *amo*A and HAC-archaeal *amo*A were determined in triplicate on the non-diluted sample. The ‘total’ archaeal *amo*A gene abundance was calculated as the sum of LAC- and HAC-archaeal *amo*A gene abundance. The reaction mixture (10 µL) contained 1x LightCycler 480 DNA SYBRGreen I Master (Roche), 0.2 µM of primers, 1 µL of DNA extract and was made-up to 10µL with PCR-grade water (Roche). All reactions were performed in 96-well q-PCR plates (Roche) with optical tape. Accumulation of newly amplified double stranded gene products was followed online as the increase of fluorescence due to the binding of the fluorescent dye SYBRGreen^®^. Specificity of the q-PCR reaction was tested on agarose gel electrophoresis and with a melting curve analysis (65-95°C) in order to identify unspecific PCR products. Each gene fragment was detected using a standard for the specific quantification of MCGI 16S rRNA gene, LAC-archaeal *amo*A and HAC-*amo*A genes and primer combinations and annealing temperature as detailed in Sintes et al. (2013). Thermocycling was performed as follows: initial denaturation at 95°C for 10 min; amplification: 50 cycles, at 95°C for 5 s, primer annealing temperature for 5 s (61ºC for 16S rRNA, 59ºC for both *amo*A genes), and extension at 72°C for 15 s, 80°C for 3 s, with a plate read between each cycle; melting curve 65 – 95°C with a read every 0.2°C held for 1 s between each read.

**T-RFLP of archaeal *amo*A genes**

Extracted DNA was used for archaeal ammonia oxidizing community fingerprinting by T-RFLP at 43 stations (excluding St13, 15, 16, 18, 21, 24, 27 and 30). The primers used for PCR were the primers cren amo_F, 5’ATGGTCTGGCTAAGACGMTGTA ([Hallam et al. 2006](#_ENREF_6)), labeled with FAM (carboxy-fluorescein) and amoAR, 5’ GCGGCCATCCATCTGTATGT ([Francis *et al.* 2005](#_ENREF_5)), labeled with VIC® (Applied Biosystems), targeting the total archaeal ammonia oxidizers community. Each 50 µL PCR reaction consisted of 0.2 µM of each primer, 200 µM of dNTP (Fermentas), 2µg BSA, and 1U Taq polymerase (Fermentas) and 5 µL of the corresponding PCR buffer, and 1 µL of the DNA extract, made up to 50 µL with UV-treated ultra-pure water (Sigma). Duplicate samples were amplified using an initial denaturation step at 94ºC (for 4 min), followed by 35 cycles of denaturation at 94ºC (1 min), annealing at 55ºC (for 1 min), and an extension at 72ºC (for 1 min). Cycling was completed by a final extension at 72ºC (for 30 min), followed by cooling at 4ºC until further processing. The PCR products were checked on a 2% agarose gel after staining with SYBRGold (Molecular Probes, Invitrogen, Carlsbad, CA, USA). The PCR products from the duplicates were pooled and purified with PCRExtract MiniKit (5-PRIME). FAM- and VIC-labeled PCR products were digested at 37ºC overnight. Each digest contained 200 ng of cleaned PCR product, 1U of restriction enzyme and the recommended buffer (final reaction volume 20 µL). Initially, 6 different enzymes were tested: *Hha*I, *Dde*I, *Mbo*I, *Rsa*I and *Hae*III (Amersham Biosciences, GE Healthcare, Buckinghamshire, UK). *Mbo*I, *Rsa*I and *Hae*III were chosen for the posterior analysis of samples as they targeted different clusters of AOA.

For T-RFLP analysis, 1.5 µL of product of the restriction digest was subsequently denatured in the presence of 10 µL deionized formamide (Invitrogen) at 95ºC for 3 min. Additionally, 0.3 µL LIZ1200 marker (Applied Biosystems) was added to each sample for size determination of FAM- and VIC-labeled fragments.

FAM- and VIC-labeled fragments were separated and detected with a 3130xL Genetic Analyzer capillary sequencer (Applied Biosystems, Foster City, CA, USA). Subsequently, the electropherograms were analyzed with GelComparII software (Bio-Rad Laboratories, Hercules, CA, USA). The threshold level to discriminate bands was set at 0.5% of the total peak height. The obtained matrix was analyzed by Primer software (Primer-E, Ltd, Ivybridge, UK) to determine the similarity between the different T-RFLP patterns obtained from the samples.

**Cloning, sequencing and phylogenetic analysis of archaeal *amo*A**

The full-length archaeal *amo*A from different samples (Table S2) was amplified using the primers cren amo_F ([Hallam *et al.* 2006](#_ENREF_6)) and amoAR ([Francis *et al.* 2005](#_ENREF_5)) (Table S1). Thermocycling was performed as follows: initial denaturation at 94°C for 4 min; amplification: 35 cycles, at 94°C for 1 min, 55ºC for 1 min, and extension at 72°C for 1 min, followed by a final extension step at 72ºC for 7 min and holding at 4ºC. The PCR product was purified using PCRExtract MiniKit (5-PRIME) and cloned with the TOPO-TA cloning kit ^®^ (Invitrogen) according to the manufacturer’s instructions. Clones were checked for the right insert by running the PCR product on a 2% agarose gel. Sequencing was performed by MACROGEN Europe using the M13 primers. The sequence data from a total of 971 clones were compiled using MEGA-5 software, and aligned together with environmental archaeal *amo*A sequences, and full-length sequences of *amo*A genes from *Nitrosopumilus maritimus*, *Candidatus* Nitrosoarchaeum limnia, *Candidatus* Cenarchaeum symbiosum, *Candidatus* Nitrososphaera gargensis and *Candidatus* Nitrosocaldus yellowstonii obtained from the NCBI database. Operational taxonomic units were defined as a group of sequences differing by less than 2%, resulting in 254 *amo*A sequences. Phylogenetic analyses were conducted in MEGA-5 ([Tamura *et al.* 2007](#_ENREF_16)). The evolutionary history was inferred using the Neighbor-Joining method ([Saitou & Nei 1987](#_ENREF_12)). Rarefaction analysis was performed using MOTHUR ([Schloss *et al.* 2009](#_ENREF_13)) for each sample and depth layer to compare the archaeal *amo*A-richness within each clone library. The Chao and ACE richness index and the Shannon and Simpson diversity index were also obtained for the different clone libraries using MOTHUR.

Sequence information obtained in this study has been deposited in Genbank, accession numbers KF727022-KF727275.

**Pyrosequencing**

454-pyrosequencing from archaeal *amo*A was performed on 18 samples distributed over the different oceanographic regions and depth layers (Table S1) at IMGM Laboratories GmbH (Germany) on a Roche 454 GS Junior platform based on titanium chemistry. All samples were barcoded using multiplex identifiers and sequenced together in one run. Total archaeal *amo*A was amplified using the same primers and thermocycling conditions as for the cloning, except that the template volume was increased to 2µL, and subsequently pyro-sequenced. In order to obtain full *amo*A sequences and to check for possible differences between sequenced regions, archaeal *amo*A was sequenced from both the forward and the reverse direction, due to the smaller average sequencing length (~430 bp) as compared to the amplicon length (632 bp). Raw 454 sequences (84810 sequences) were initially trimmed using Lucy 1.20 ([Chou & Holmes 2001](#_ENREF_2)) keeping sequences of ≥250 nt which had an average Phred score of ≥27. Subsequently, the remaining sequences were screened for the barcode and primer sequences keeping only the sequences that had exact matches (68517 sequences).

The sequences selected by the above procedure were processed following a similar pipeline as described elsewhere ([Pester *et al.* 2012](#_ENREF_10)). Briefly, sequences were pre-clustered using the pre.cluster function in MOTHUR ([Schloss *et al.* 2009](#_ENREF_13)) with n=3 (sequence identity ≥97.6% for sequences ≥250 nt). Representatives of the pre.cluster step were further grouped using the CD-HIT-454 (http://weizhong-lab.ucsd.edu/cd-hit/servers.php) clustering tool ([Huang *et al.* 2010](#_ENREF_8)) at a 98.5% sequence identity level over 97% of the smaller sequence. Thereafter, HMMFrame ([Zhang & Sun 2011](#_ENREF_20)) was used to screen possible frame shifts in representative sequences of all CD-HIT clusters, resulting in 6837 and 11505 representative forward and reverse sequences, respectively.

After manual chimera removal ([Pester *et al.* 2012](#_ENREF_10)), sequences were grouped based on their sequencing direction (forward or reverse) and rarefaction curves, binning into OTUs, and α-diversity analysis were conducted using MOTHUR ([Schloss *et al.* 2009](#_ENREF_13)). OTUs were assigned as those sequences differing ≤ 2%. The remaining sequences were aligned together with the 254 clone sequences and NCBI reference sequences from *N. maritimus*, *Nitrososphaera gargensis*, *Nitrosoarchaeum limnia*, and *Cenarchaeum symbiosum* to infer their phylogeny. Raw 454-pyrosequences of *amo*A have been deposited in NCBI, accession number SRP049002.

**Statistics**

Statistical analyses were performed with Primer 6.1.7 software (Primer-E, Ltd) and SigmaPlot 11 (Systat Software Inc.). Whole communities were compared by calculating the Bray-Curtis index of similarity, which considers the relative contribution of each OTU to the total OTUs amplified DNA. Bray-Curtis index of similarity was calculated using Primer software. The resulting matrixes were subjected to cluster-analysis via the unweighted pair-group method using mean average (UPGMA) ([Sokal & Rohlf 1995](#_ENREF_15)). The Bray-Curtis index was also used to assess the similarity between the community composition of different samples. Polynomial regression was used to inspect the relationship between the similarity in community composition and the distance between samples through the Atlantic Ocean for different depth layers. Since the data used for this analysis consist of pairwise comparisons, thus lacking independence, bootstrapping (10,000 replications) was used to test whether the slopes of the polynomial regression obtained were different from zero ([Efron & Tibshirani 1993](#_ENREF_4); [Horner-Devine *et al.* 2004](#_ENREF_7)) for the different depth layers. Only the equation obtained for the lower bathypelagic was not significantly different from zero (p>0.4).

Two-way analysis of similarities was used to test for significance of the depth-related and the regional distribution of the archaeal ammonia oxidizing community. Grouping of samples was done according to the previously described oceanographic regions and depth layers (epipelagic: 50m depth, mesopelagic: 200-1000m depth, upper-bathypelagic: 1000-2000m depth, lower-bathypelagic: >2000m depth).

Canonical correspondence analysis (CCA) was conducted on XLStat to relate the abundance of OTUs (from T-RFLP fingerprints and from 454-pyrosequencing libraries) to the environmental variables ([ter Braak 1986](#_ENREF_17)).

Partial RDA was used as described elsewhere ([Liu 1997](#_ENREF_9)) to partition the variation of the AOA community composition explained by environmental, spatial and temporal factors. OTU abundance data obtained from T-RFLP fingerprinting was normalized and standardized and used as response variable. To avoid co-linearity among variables within each category, explanatory variables with the highest variance inflation factor (VIF) were sequentially removed until all VIF were smaller than 20 ([ter Braak & Smilauer 2002](#_ENREF_18)). Finally, six environmental parameters (temperature, salinity, dissolved oxygen concentration, fluorescence, silicate and nitrite), two spatial (region: ARCT, NADR, NAG, WTRA, SATL, SANT, and depth layer: epi-, meso-, upper and lower bathypelagic) and one temporal (month) were selected to perform the variation partitioning analysis.

**REFERENCES**

Agogué H, Brink M, Dinasquet J, Herndl GJ (2008) Major gradients in putatively nitrifying and non-nitrifying Archaea in the deep North Atlantic. *Nature* **456**, 788-791.

Chou HH, Holmes MH (2001) DNA sequence quality trimming and vector removal. *Bioinformatics* **17**, 1093-1104.

De Corte D, Sintes E, Yokokawa T, Reinthaler T, Herndl GJ (2012) Links between viruses and prokaryotes throughout the water column along a North Atlantic latitudinal transect. *Isme Journal* **6**, 1566-1577.

Efron B, Tibshirani R (1993) *An Introduction to the Bootstrap* Chapman and Hall, New York, USA.

Francis CA, Roberts KJ, Beman JM, Santoro AE, Oakley BB (2005) Ubiquity and diversity of ammonia-oxidizing Archaea in water columns and sediments of the ocean. *Proceedings of the National Academy of Sciences of the United States of America* **102**, 14683-14688.

Hallam SJ, Konstantinidis KT, Putnam N*, et al.* (2006) Genomic analysis of the uncultivated marine Crenarchaeote Cenarchaeum symbiosum. *Proceedings of the National Academy of Sciences of the United States of America* **103**, 18296-18301.

Horner-Devine MC, Lage M, Hughes JB, Bohannan BJM (2004) A taxa-area relationship for bacteria. *Nature* **432**, 750-753.

Huang Y, Niu B, Gao Y, Fu L, Li W (2010) CD-HIT Suite: a web server for clustering and comparing biological sequences. *Bioinformatics* **26**, 680-682.

Liu Q (1997) Variation partitioning by partial redundancy analysis (RDA). *Environmetrics* **8**, 75-85.

Pester M, Rattei T, Flechl S*, et al.* (2012) amoA-based consensus phylogeny of ammonia-oxidizing archaea and deep sequencing of amoA genes from soils of four different geographic regions. *Environmental Microbiology* **14**, 525-539.

Reinthaler T, Sintes E, Herndl GJ (2008) Dissolved organic matter and bacterial production and respiration in the sea-surface microlayer of the open Atlantic and the western Mediterranean Sea. *Limnology and Oceanography* **53**, 122-136.

Saitou N, Nei M (1987) The neighbor-joining method - a new method for reconstructing phylogenetic trees. *Molecular Biology and Evolution* **4**, 406-425.

Schloss PD, Westcott SL, Ryabin T*, et al.* (2009) Introducing mothur: Open-Source, Platform-Independent, Community-Supported Software for Describing and Comparing Microbial Communities. *Applied and Environmental Microbiology* **75**, 7537-7541.

Sintes E, Bergauer K, De Corte D, Yokokawa T, Herndl GJ (2013) Archaeal amoA gene diversity points to distinct biogeography of ammonia-oxidizing Crenarchaeota in the ocean. *Environmental Microbiology* **15**, 1647–1658.

Sokal RR, Rohlf FJ (1995) *Biometry. The principles and practice of statistics in biological research* W. H. Freeman, New York.

Tamura K, Dudley J, Nei M, Kumar S (2007) MEGA4: Molecular Evolutionary Genetics Analysis (MEGA) software version 4.0. *Molecular Biology and Evolution* **24**, 1596-1599.

ter Braak CJF (1986) Canonical correspondence analysis - a new eigenvector technique for multivariate direct gradient analysis. *Ecology* **67**, 1167-1179.

ter Braak CJF, Smilauer P (2002) *CANOCO Reference manual and Canodraw for Windows user's guide. Software for canonical community ordination (version 4.5).* Microcomputer power, Itaca, New York.

Wuchter C, Abbas B, Coolen MJL*, et al.* (2006) Archaeal nitrification in the ocean. *Proceedings of the National Academy of Sciences of the United States of America* **103**, 12317-12322.

Zhang Y, Sun Y (2011) HMM-FRAME: accurate protein domain classification for metagenomic sequences containing frameshift errors. *Bmc Bioinformatics* **12**, 198.
